# MuRF1/TRIM63, Master Regulator of Muscle Mass

**DOI:** 10.3390/ijms21186663

**Published:** 2020-09-11

**Authors:** Dulce Peris-Moreno, Daniel Taillandier, Cécile Polge

**Affiliations:** INRA, UNH, Unité de Nutrition Humaine, Université Clermont Auvergne, F-63000 Clermont-Ferrand, France; dulce.peris-moreno@inrae.fr (D.P.-M.); daniel.taillandier@inrae.fr (D.T.)

**Keywords:** MuRF1/TRIM63, TRIM E3 ligase, skeletal muscle, heart, atrophy, hypertrophy, cardiomyopathy, contractile proteins, pharmacological inhibitors, chronic diseases

## Abstract

The E3 ubiquitin ligase MuRF1/TRIM63 was identified 20 years ago and suspected to play important roles during skeletal muscle atrophy. Since then, numerous studies have been conducted to decipher the roles, molecular mechanisms and regulation of this enzyme. This revealed that MuRF1 is an important player in the skeletal muscle atrophy process occurring during catabolic states, making MuRF1 a prime candidate for pharmacological treatments against muscle wasting. Indeed, muscle wasting is an associated event of several diseases (e.g., cancer, sepsis, diabetes, renal failure, etc.) and negatively impacts the prognosis of patients, which has stimulated the search for MuRF1 inhibitory molecules. However, studies on MuRF1 cardiac functions revealed that MuRF1 is also cardioprotective, revealing a yin and yang role of MuRF1, being detrimental in skeletal muscle and beneficial in the heart. This review discusses data obtained on MuRF1, both in skeletal and cardiac muscles, over the past 20 years, regarding the structure, the regulation, the location and the different functions identified, and the first inhibitors reported, and aim to draw the picture of what is known about MuRF1. The review also discusses important MuRF1 characteristics to consider for the design of future drugs to maintain skeletal muscle mass in patients with different pathologies.

## 1. Introduction

Skeletal muscle atrophy occurs during physiological (e.g., bed rest) and pathological (diabetes, cancer, renal failure, sepsis, etc.) conditions. In the latter case, muscle loss impairs the healthiness of patients, their ability to respond to treatments and increases mortality. It is now admitted that an increased proteolysis is the main component of muscle wasting, and that the ubiquitin-proteasome system (UPS) and the autophagy proteolytic systems are the main systems involved. The UPS is believed to be the main actor of the degradation of contractile proteins, the most abundant proteins in muscle. A potential strategy to improve patients’ condition is thus to reduce muscle wasting by regulating the UPS.

The proteins to be degraded by the 26S proteasome are labelled by a post-translational modification called polyubiquitination (polyUb) that results from the sequential action of three enzymes: The ubiquitin-activating enzyme E1, ubiquitin-conjugating enzymes E2 (around 40 in mammals), and ubiquitin ligase E3 (>600 in mammals) (for a review see [1]).

Muscle-specific RING finger protein 1 (MuRF1), also named TRIM63, is a RING-type E3 ligase that aroused growing interest in the context of muscle atrophy. MuRF1 was first described by Centner et al. [2] in an attempt to identify myofibrillar proteins interacting with the kinase domain of titin, a large myofibrillar protein located in the sarcomere [2]. Soon after, MuRF1 was described as a player in skeletal muscle atrophy thanks to transcriptome analysis in rats subjected to denervation, immobilization, or unweighting atrophy [3]. Among the genes array, MuRF1/TRIM63 appeared to be systematically up-regulated in the three models. Further, in vitro experiments identified MuRF1/TRIM63 as a RING-type E3 ubiquitin ligase whereas in vivo experiments using Knock-Out (KO) mice models established this enzyme as a marker for multiple models of skeletal muscle atrophy [3]. Subsequent work has identified more than 25 atrophying situations where MuRF1 appeared to be up-regulated. MuRF1 is one of the founding members of the so-called “atrophy-related genes” (atrogenes) [4].

In the past 20 years, MuRF1 has been intensively studied due to its potential role in the regulation of muscle atrophy under catabolic situations notably within several diseases. The importance of MuRF1 in the protection of cardiac muscle is also an important field of research. This review provides an update on the research performed on MuRF1, in skeletal and cardiac muscles. The aim is to draw a clear and accurate picture of what is known about MuRF1.

## 2. Structure

### 2.1. MuRF1 Belongs to the Tripartite Motif (TRIM) Family of Proteins

MuRF1 belongs to the RING-B-box-coiled-coil (RBCC) or tripartite motif (TRIM) family of proteins which includes more than 70 proteins in humans. This family is characterized by the presence of three conserved zinc-binding domains (RING finger, a B-box type 1, and/or B-box type 2) followed by a coiled-coil (CC) region [5,6]. These three motifs, located at the N-terminal end of the protein, have been selectively conserved across evolution and constitute an integrated functional structure [7]. Conversely, the C-terminal end presents variable domains (e.g., AT, COS-box, Filamin, or PRY-SPRY), conferring specific physiological functions to each TRIM member [8,9,10]. Such a domain diversity within the C-terminal has been used as a main trait for the different classifications reported so far within this family [8,11,12,13,14]. Notably, not all the TRIM proteins contain a B-box type 1 domain or a RING-finger domain [6].

TRIM proteins are implicated in a myriad of cellular processes thanks to their E3 ubiquitin-ligase signature mediated by their RBCC motif [6,15]. Indeed, they constitute the largest family of RING E3-ligases and are implicated in a variety of human pathologies [11,16]. Among them, MuRF1 is one of the most studied proteins due to its implication in muscle remodeling.

**Figure 1:**
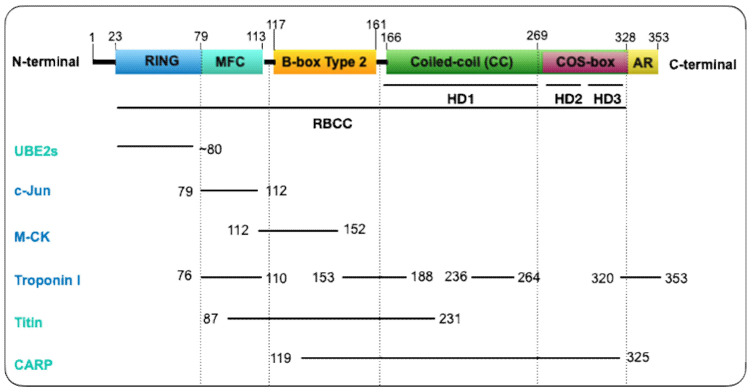
MuRF1 structure and known regions of interaction with some partners. MuRF1 structure is characterized by a RBCC (RING-B-box-coiled-coil) motif constituted by an N-terminal RING domain (23–79), a MuRF family specific motif (MFC) motif (79–113), a B-box type 2 (Bb2) domain (117–161), a central helical domain (166–328) with a COS-box motif (269–328) and a C-terminal acidic tail (328–353). Several regions have been identified for their interaction with MuRF1 partners (substrates in dark blue and other interactors in light blue): Titin [2], cardiac troponin I [21,28], muscle-type creatine kinase (MCK) [29], c-Jun [22], the ubiquitin conjugating enzymes (UBE2s) [30], CARP 119–325 [31]. HD, helical domain.

MuRF1 belongs to the Class II of TRIM proteins and is characterized by the presence of a MuRF family specific motif (MFC) and solely a B-box type 2 (Bb2) domain within the constant RBCC motif, a COS-box, and an acidic tail motif (AT) in the C-terminal region (Figure 1) [17]. The RING domain (C3HC4 zinc finger-type [18]), although not crystalized yet, is assumed (based on other TRIMs’ structures) to present a conserved docking region for the E2-ubiquitin conjugating enzymes, endowing the E3 ubiquitin-protein ligase activity [9,16,19,20]. The MFC is a 35 residues motif specific for the MuRF family located between residues 79 and 113 (Figure 1). It has been implicated in the interaction with different substrates or partners (troponin I, c-Jun and titin A168-A170 region) [2,21,22] (Figure 1). Similarly, the Bb2 domain interacts with muscle-type creatine kinase, titin, and CARP. The Bb2 domain also provides stability to the CC domain [23]. This is possible thanks to its quaternary structure arrangement [17,23,24]. Such an organization has also been observed in TRIM5α [25] and TRIM27 [26], that possesses a Bb2 domain that self-associates, which may contribute to enhanced E3 ligase activity. The CC domain, on the other hand, and by contrast with other TRIM proteins, does not orchestrate MuRF1′s oligomerization. The capping by the flanking motifs (Bb2 and COS-box) controls the CC assembly and thus the quaternary structure of MuRF1 into an antiparallel dimer in solution [27]. Specifically, the COS-box is the motif required to achieve a productive homodimerization and for the recruitment of MuRF1 to the sarcomere [24,27]. Lastly, the C-terminal domain, an acidic tail (AT), is predicted to be unstructured [23].

### 2.2. What Is Known about the Tridimensional Structure of MuRF1?

Little is known about MuRF1 tridimensional (3D) configuration due to the complexity for crystallizing its different domains. The crystal structure of the RING domain has not been determined yet due to its instability. However, based on other TRIM proteins analyses, the conserved pattern of cysteine and histidine residues in MuRF1 might be buried within the interior of the domain. Such conformation of the RING domain coordinates two zinc ions in a cross-braced fashion and helps maintain the overall structure [7,15]. In addition, preferences for specific residues in determined positions may underlie binding specificity towards different Ub-conjugating E2 enzymes [11].

The Bb2 and CC structures of MuRF1 have been successfully characterized. Mrosek and collaborators [17] showed by X-ray crystallography and Nuclear Magnetic Resonance (NMR) spectroscopy that the Bb2 domain of MuRF1 in humans contains three loops and coordinates two zinc ions in a cross-brace structure. In addition, a stable self-association of Bb2 was described at low concentrations, indicating a possible role in the overall assembly of MuRF1. This was further suggested by light scattering assays where the Bb2 domain proved to be necessary for high-order association for the Bb2CC region of the protein. Besides, the lack of a defined hydrophobic core within the Bb2 domain reinforces the idea that this domain is mostly stabilized by self-assembly events and zinc binding [17].

The 3D structure of the coiled-coil domain of MuRF1 revealed that the helical domain (HD) is sub-divided in three portions (HD1, HD2, and HD3) according to the three distinct helical regions [27]. The C-terminal of the HD1 is characterized by a CC formation and HD2 and HD3 correspond to the COS-box motif (Figure 1). The crystal structure of the human MuRF1-CC segment turned out to be a tetrameric palindromic structure [27]. However, crystallization of the HD1 together with its flanking motifs (Bb2 and COS-box) revealed that the ground association state of this complex was in fact a dimer and micrographs showed a rod-like morphology with Bb2 in apical position. Then, ab initio models found that the other two HD regions (COS-box motif) formed a compact arrangement that resembled a minimal spectrin fold against the central HD1, and size-exclusion chromatography confirmed the interaction between HD1 and the COS-box motif. Therefore, the interaction between HD1 and its flanking motifs prevented self-association into non-native high assemblies, suggesting that both Bb2 and COS-box act as terminal clamps that secure the correct self-assembly of the CC [27]. Very recently, Stevens et al. (2019) deciphered the orientation of MuRF1 oligomer by mutating different cysteine residues within the HD1 [23]. The different residues were selected according to previously characterized TRIM protein structures. Electron paramagnetic resonance (EPR) analyses revealed an antiparallel arrangement of the MuRF1-HD1 dimer with the COS-box motif packing against the coiled-coil region. Such an arrangement of COS-box confirmed MuRF1 as a canonical model of TRIM fold protein [23].

### 2.3. Two Other MuRF Proteins

MuRF1 belongs to a small family with three members in mammals, namely MuRF1/TRIM63, MuRF2/TRIM55, and MuRF3/TRIM54. They harbor a similar structure: N-terminal RING, MFC and B-box domains, two central coiled-coil motifs, and a C-terminal acidic tail domain [2]. Within the different MuRF members, the best conserved region is the MFC whereas the C-terminal acidic tail domain is the most divergent and subjected to differential splicing events. Indeed, while a single protein is produced for MuRF1, several isoforms have been found for MuRF2 and MuRF3. Despite its variability, the acidic tail domain is also characterized by a high content of glutamic and aspartic amino residues in MuRF1-3 [2]. The different MuRF proteins can interact to form heterodimer [2].

#### 2.3.1. MuRF2

MuRF2 shares 62% homology with MuRF1 [2]. Four different splicing variants have been described for MuRF2 (p60A, p60B, p50A, p27A) in mice and humans [32]. Regarding their structure, they share the common N-terminal RING and B-box domains and the C-terminal part. These three domains constitute the p27A isoform, the shortest isoform of MuRF2. On the other hand, p50 and p60A isoforms, although generated by sequential internal expansion as p27A, present a coiled-coil domain and a glutamine-rich domain. In addition, p60A presents a serine/alanine-rich domain. The fourth isoform, p60B, has a different origin, being generated by the splicing of an additional exon that leads to the generation of an alternative C-terminus [33]. The variants are differentially expressed throughout development and in a tissue-specific manner. In skeletal muscle, the p50A isoform of MuRF2 is predominant in embryonic stages whereas p60A is mostly present in postnatal stages and p27A is heart-specific [32].

#### 2.3.2. MuRF3

MuRF3 was historically the first member of the MuRF family discovered and was referred to as MuRF [24]. One year later, Centner et al. [2] discovered MuRF1 and MuRF2 and coined a new name to Spencer’s protein, as MuRF3. MuRF3 structure is similar to MuRF1 and MuRF2 and is specifically expressed in cardiac and skeletal muscle cells throughout perinatal development [24]. MuRF3-silencing experiments proved that MuRF3 plays an essential role in the myogenic differentiation and myotube formation processes by regulating the expression of MHC, MyoD, and myogenin and stabilizing microtubules [24]. MuRF3 shares 77% of homology with MuRF1 and 65% with MuRF2 [2]. Additionally, one variant has been identified for MuRF3 in mice and humans [32].

## 3. Regulation of MuRF1

### 3.1. Transcriptional Regulation

MuRF1 is present in smooth, cardiac, and skeletal muscles but most of the data about its transcriptional regulation have been obtained in skeletal muscles. Thus, unless stated, this paragraph is valid for skeletal muscle only.

As already mentioned, MuRF1 is part of a three-member family in mammals, each MuRF being differentially expressed during the development. In skeletal muscles, MuRF1 is hardly detectable during embryogenesis and its expression is moderately increased after birth or during myoblast fusion [32]. This is in sharp contrast with MuRF2 and MuRF3, which exhibit strong and developmental-related expression during embryogenesis and after birth, respectively [32]. MuRF1 is expressed at low levels in adult muscles in normal conditions but is highly over-expressed at the mRNA levels upon catabolic stimuli [4]. The protein expression generally parallels the mRNA content. This induction of expression is specific of MuRF1 as neither MuRF2 nor MuRF3 are considered as muscle-atrophy specific genes [4,34] even though recent work found that MuRF2 is over-expressed in the soleus muscle from hypertension mice [35]. This implies that MuRF1 is required for the development of muscle atrophy and that MuRF1 levels are limiting and necessitate de novo synthesis. It soon became evident that MuRF1 was dramatically up-regulated upon most if not all atrophy situations [3,36,37] and it is now widely accepted that MuRF1 transcription is tightly linked to the development of the atrophy program in both rodent models and human patients [4].

MuRF1, together with other genes, is one of the founding members of a gene family considered as the hallmark of muscle atrophy, namely the atrogenes (see [4] for a recent review). As expected for a gene specific of atrophy program, MuRF1 is readily down regulated upon recovery from muscle atrophy. This has been demonstrated in hindlimb suspended rats, where MuRF1 returns to basal levels within less than 18 h, in good coordination with other atrogenes [38]. Such a down-regulation was also observed using other reversible rodent atrophy models like nerve injury [39,40] or tetrodotoxin injection [41]. Accordingly, anabolic effectors like leucine [42,43,44], insulin, or IGF-1 [45,46,47,48] are able to blunt the expression of MuRF1 in parallel to muscle sparing during different catabolic situations (dexamethasone, unloading, burns). However, IGF-1 is not able to repress Angiotensin II-induced [49] or LPS-induced [50] muscle atrophy, suggesting that the prevalence of anabolic and catabolic stimuli depends on the nature of the stimuli.

**Figure 2:**
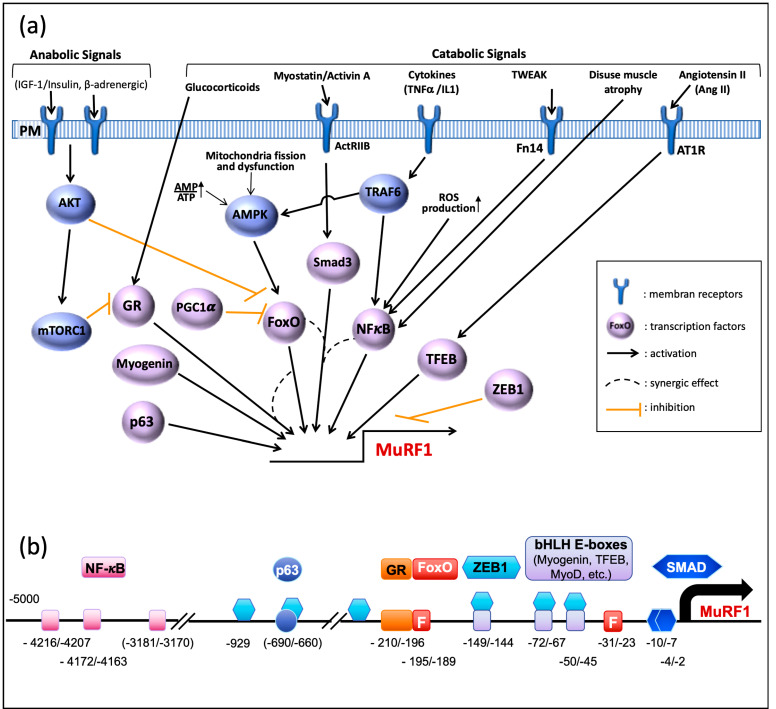
Transcriptional Regulation of MuRF1. (**a**) Pathways that control MuRF1 transcription. MuRF1 transcription is regulated by different stimuli that activate several signaling pathways, and several different transcription activators and repressors. See main text for further details. GR, glucocorticoid receptor, PM, plasma membrane. (**b**) The promoter of the mouse (C57BL/6J) MuRF1 gene is schematically shown with the transcription factors potentially promoting or interfering with MuRF1 transcription in the skeletal muscle. Binding sites are numbered starting with the transcription initiation as base +1. Only the sites experimentally tested are shown but several other potential sites exist in the promoter sequence for most of them. For the repressor Zeb1, 30 other sites exist but they have not been individually tested and only the ones present in the proximal region of the promoter are shown. In the heart, the activity of a MEF2 binding site (−32/−23) encompassing the proximal FoxO site was reported.

Several signaling pathways are involved in the control of MuRF1 expression (Figure 2a). During catabolic situations, increased catabolic signals (myostatin, glucocorticoids, cytokines) or repressed anabolic ones (insulin/IGF-I) induce the expression of MuRF1, which can be either direct or not (see [51] for a review). Accordingly, several transcription factors directly drive the MuRF1 promoter (Figure 2b), the FoxO family being considered as a master regulator of this E3 ligase. However, the role of each transcription factor is context-dependent and can be either crucial or negligible depending on the catabolic situation.

**FoxO family.** Pioneering studies found that FoxO1 and FoxO3a are up-regulated upon several atrophy situations, like fasting and Dexamethasone (Dex) treatment [52,53]. In line with these studies, compelling data pointed out that FoxO1 and FoxO3a are the main regulators of MuRF1 expression and hence of muscle mass.

Upon Dex treatment, FoxO1 is able to bind MuRF1 promoter in cultured myotubes and Dex-treated mice, and multimerization seems necessary for achieving optimal transcription of MuRF1 [54]. Upon glucocorticoid treatment (Dex), the overactivity of the MuRF1 promoter is synergized by the concomitant binding of the glucocorticoid receptor (GR). This was specific of FoxO1 as FoxO3a and FoxO4 were neither able to activate MuRF1 promoter nor to potentiate GR upon Dex treatment [54]. Surprisingly, FoxO3a was even inhibitory against GR. Despite the presence of several putative FoxO binding elements in the MuRF1 promoter, mutation of the site next to the GR element completely abolishes FoxO1 activity [54] (Figure 2b). Similarly, the increased expression of FoxO1 upon sepsis and its subsequent DNA binding activity was controlled by the GR activity (by contrast with FoxO3a), further suggesting a tight collaboration between these GR and FoxO1 [55]. Surprisingly FoxO1-dependent MuRF1 expression may also be achieved without binding to the promoter although this was limited to in vitro studies [56]. Finally, muscle atrophy induced by Dex in cultured cells or by chronic kidney disease in mice was attributed to FoxO1 with an induction of MuRF1 and MAFbx/Atrogin-1 [57].

FoxO3a increases MuRF1 expression upon serum deprivation and SMAD3 cooperates with FoxO3a for enhancing MuRF1 expression. Despite the presence of SMAD binding elements in the MuRF1 promoter (Figure 2b), SMAD3 alone is unable to activate MuRF1 transcription and can be considered as a co-activator [58,59]. The involvement of FoxO3a in controlling MuRF1 promoter was also found using a model of Cushing Syndrome in rats by infusion of adrenocorticotropic hormone (ACTH). Such a model induces an overactivation of the GR and further induced FoxO3a enrichment in the promoter of MuRF1 [60]. By contrast with the short-term Dex-treatment models that found an exclusive role of FoxO1 [54,57], four-weeks ACTH infusion induced MuRF1 expression, which was exclusively controlled by FoxO3a. Interestingly, the expression of FoxO3a was highly dependent on the GR as its expression was depressed by the RU486 GR antagonist. Finally, using mice KO for each FoxO member, it was shown that FoxO3a was necessary for optimal expression of MuRF1 in denervated and fasted animals [61]. Altogether, these studies highly suggest that both FoxO1 and FoxO3a are potent inducers of MuRF1 in several atrophy models, but that their implication may be highly dependent on the model used and synergized by other transcription factors.

So far, FoxO4 is the family member dispensable for expressing most atrogenes, including MuRF1, and promoting muscle atrophy [54,60,61]. Indeed, while FoxO4 may be able to bind MuRF1 promoter, only few atrogenes can be controlled by FoxO4 like MAFbx/Atrogin-1 in a TNF-induced muscle atrophy model [62] or Gadd45 in denervated and fasted animals [61].

Are the FoxOs redundant or do they have specific roles? To answer this question, several studies used FoxO triple KO animals. In mice, FoxO1/3a/4 knockout totally protected skeletal muscles from diabetes-induced atrophy [63]. The authors found that the presence of FoxOs was necessary and sufficient for both blunting insulin signaling (PI3K/Akt) and stimulating the transcription of MuRF1 and other atrogenes. Similarly, using single and triple KO of FoxO genes, Pr. Sandri’s laboratory showed that FoxO3a was the only FoxO that partially protected from fasting or denervation-induced muscle atrophy but MuRF1 induction was modestly reduced, suggesting a redundancy by other FoxOs [61]. Interestingly, FoxO1 was necessary for optimal overexpression of MuRF1 in these catabolic situations, which altogether implies that at least FoxO1 and 3a functions are at least partly redundant.

**PGC1-alpha** is a transcriptional co-activator known to interact with FoxO1 in liver, which promotes the transcription of genes important for gluconeogenesis [64]. By contrast, in skeletal muscle, PGC1-alpha negatively regulates FoxO3a-induced skeletal muscle atrophy in denervated or fasted mice [65]. Although direct implication of PGC1-alpha in the control of MuRF1 transcription is still lacking, PGC1-alpha over expression counteracts MuRF1 up-regulation (Figure 2a).

**Glucocorticoid receptor (GR)**. A GR binding sequence is present in the MuRF1 promoter and GR efficiently binds to the MuRF1 promoter upon Dex treatment [54]. However, a homo-dimerization mutant of the GR element altered GR binding while no effect was observed on Dex-induced skeletal muscle atrophy [54]. By contrast, skeletal muscles from mice knocked out for the GR element lost their capacity to increase MuRF1 levels upon Dex treatment and were protected from Dex-induced atrophy (but not from nerve lesion or hindlimb suspension) [66] [Watson, AJP 2012]. This means that the mechanisms by which the GR controls muscle mass via MuRF1 expression need further investigations.

**The NF-kB signaling pathway** is activated upon inflammatory cytokine production and the MuRF1 promoter possesses NF-kB binding sites (Figure 2b), which enables a direct induction of MuRF1 mRNA production [67] (Figure 2a). The NF-kB pathway can be activated by TNFα or by TWEAK, two inflammatory cytokines that potently induce MuRF1 overexpression and muscle atrophy [68,69]. As an example of the respective roles of the different transcription factors, the NF-kB sites were required for disuse atrophy-linked MuRF1 expression, while FoxO sites were dispensable [67]. A similar conclusion was obtained using a model of acute lung injury where all the NF-kB binding elements were removed from the MuRF1 promoter [70]. Muscle wasting can also occur in situations of oxidative stress due to abnormal ROS production and the up-regulation of MuRF1 is driven by NF-kB but the presence of SMAD3 is mandatory [71]. In absence of SMAD3, the transcription factor CHOP is able to bind the MuRF1 promoter and to induce an increased muscle proteolysis [71]. However, this was determined in Smad3-null mice that lacked the NF-kB response and the role of CHOP in wild-type animals awaits more studies.

**The p53 family** of transcription factors is implicated in a multitude of cellular processes, including organ development [72], neuronal apoptosis [73], and tumor suppression [74]. Intriguingly, among p53 family, p63/Tap63 is also implicated in the development of muscle atrophy during amyotrophic lateral sclerosis (ALS) or denervation in human or mouse models [75]. Interestingly, p63 directly binds to MuRF1 promoter in C2C12 cells, indicating a direct action of this transcription factor. Whether p63 cooperates with other transcription factors to stimulate MuRF1 transcription remain to be discovered.

**TFEB** belongs to the bHLH family of transcription factors implicated in the control of autophagy genes [76,77]. The MuRF1 promoter possesses binding elements for TFEB, which is implicated in angiotensin II-induced muscle atrophy [78] (Figure 2a,b). Angiotensin II (Ang II) is considered as a signaling element of muscle atrophy induced upon chronic cardiac failure. Accordingly, direct infusion of Ang II in mice induces muscle atrophy and an overexpression of MuRF1 [49,78]. Additionally, while IGF-1 was able to counteract Ang II-regulated MAFbx expression via Akt-dependent FoxO1 repression, IGF-1 was unable to abolish MuRF1 up regulation [49], thus suggesting prevalence of TFEB in controlling MuRF1 expression in Ang II-dependent skeletal muscle atrophy.

**Myogenin** is, like TFEB, a member of the bHLH transcription factors family essential for myogenesis and skeletal muscle cell differentiation [79,80] and it has also been described as an important modulator of MuRF1 expression in denervated muscles [81]. Likewise, myogenin shares with TFEB the same three boxes found in the MuRF1 promoter and close to FoxO binding sites [81] (Figure 2). Whether a cooperation or a synergy exists between myogenin and FoxOs and what the prevalence is between TFEB and myogenin are still open questions.

**Zeb1** is a transcription repressor mostly known for its role in cancer progression. However, Zeb1 has been recently identified as a negative regulator of the expression of MuRF1 and several other atrogenes [82]. Like myogenin and TFEB transcription factors, Zeb1 recognizes E-box motifs (CANGTG) but also E-box-like motifs (CANNTG). Zeb1 can directly bind to the MuRF1 promoter in transfected cells and is able to repress MuRF1 expression. This negative control of MuRF1 promoter is (at least in part) dependent on the binding of the co-repressor Ctbt to Zeb1. Overexpression of exogenous Zeb1 is also able to counteract FoxO3a transcriptional activity, which suggests that its binding partially impedes FoxO3a binding. Interestingly, Zeb1 efficiently binds the promoter of MuRF1 in specific stages, i.e., in myoblast and in atrophying myotubes, and may also be displaced by MyoD1; however, the latter has only been demonstrated for MAFbx/Atrogin-1 [82].

**C/EBP** (CCAAT/enhancer binding protein) is a family of transcription factors whose expression is mainly linked to cell proliferation and differentiation [83]. Like for several genes belonging to the UPS, the MuRF1 promoter possesses putative binding sites for C/EBP transcription factors but, so far, no study has shown physical interaction between C/EBP and the MuRF1 promoter (Figure 2b). Using siRNA approaches, it was shown that C/EBPbeta was implicated (directly or indirectly) in the up-regulation of MuRF1 in Dex-treated muscle cells [84]. However, the role of C/EBPbeta was partial and limited to undifferentiated myoblasts treated with Dex, the expression of MuRF1 (and MAFbx) being unaffected by C/EBP beta in non-catabolic muscle cells. Combined with the known role of C/EBP transcription factors during cellular proliferation and differentiation, this suggests that the role of C/EBPbeta may be limited to specific stages of development. Similarly, the elevated transcription of MuRF1 was independent of C/EBPbeta in mice subjected to myostatin-induced muscle atrophy, by contrast with other atrogenes [85]. C/EBPdelta, another member of the family, was also found to activate the MuRF1 promoter in C2C12 cells, although modestly when compared to MAFbx [86]. Intriguingly, Stat3 blunted C/EBPdelta action in C2C12 myotubes for both MuRF1 and MAFbx, whereas both Stat3 and C/EBPdelta seemed necessary for the development of cancer-linked muscle atrophy. Altogether, the exact role of C/EBP transcription factors on MuRF1 transcription in vivo is still poorly understood and needs further work.

Few data were obtained in the heart or by using cultured cardiac cells and differential role of the transcription factors may characterize cardiac and skeletal muscle control of MuRF1. However, high fat-induced obesity induces cardiac hypertrophy through repressed FoxO3a activity and consequently lower MuRF1 transcription levels [87], which suggests an important role of FoxOs in the heart. Likewise, FoxO3a is also implicated in the control of MuRF1 transcription upon abnormal Ca^2+^ homeostasis, FoxO3a being controlled by calcineurin [88]. FoxO1 and 3a were also proposed to be important drivers of cardiac atrophy upon sympathetic denervation although direct effect was not addressed (Giulia, Circ Res, 2013).

Besides all the transcription factors identified within skeletal muscles, MEF2 was shown to modulate MuRF1 transcription in the heart [89]. The authors found that nutrient deprivation enhanced the activity of MEF2 that was itself controlled by AMPK. The binding sequence of MEF2 overlaps the proximal FoxO site of the MuRF1 promoter, suggesting that these transcription factors may be alternatively active depending on the upstream stimuli. Indeed, using siRNA approaches, AMPK was also proposed as a main driver of FoxO3a during skeletal muscle atrophy provoked by hypercapnia in skeletal muscle [90]. It remains to be determined whether MEF2 and FoxO3a are specific of the muscle type (cardiac vs. skeletal) and whether they collaborate or compete.

### 3.2. Post-Translational Modifications

Like most proteins, MuRF1 may be subjected to post-translational modifications for modifying its activity or location. Phosphorylation is one of the most common post translational modifications altering protein fate in cells. However, despite the presence of two clusters of residues potentially phosphorylated (https://www.phosphosite.org/), no experimental evidence has been found for the phosphorylation of MuRF1.

The only post-translational modification described so far for MuRF1 is the SUMOylation of a lysine residue. SUMO is a Ubiquitin-like modifier that generally does not target proteins for degradation but rather modify their activity or their location, and three SUMO isoforms exist in mammalian cells. A first evidence for MuRF1 SUMOylation came from mechanically ventilated diaphragm in which MuRF1^-SUMO1^ was identified by mass spectrometry (potentially in K206, K280, and K297) and the potential SUMOylation was confirmed by an in vitro assay [91]. More recently, the same team identified K238 as the SUMO1 site and found that SUMOylation was induced by ROS production [92]. The role of sumoylation of MuRF1 is far from being understood even though the authors concluded it might lead to MuRF1 localization.

Ubiquitination is not restricted to extra-UPS targets and numerous UPS enzymes are themselves ubiquitinated. The auto-ubiquitination of several E3 ligases has been described, but most studies used in vitro assays and need further validation in vivo for confirming a physiological role of polyUb. MuRF1 auto-ubiquitination is thus largely used in in vitro assay [93,94] but only a single study described MuRF1 ubiquitination in a cellular context [95] and found MuRF1^-Ub^ in peripheral blood. The signals obtained were compatible with mono, multi-mono, or short polyUb chains, which may not be a degradation signal. More work is clearly needed for clarifying the presence and role of MuRF1^-Ub^ in striated muscles.

## 4. MuRF1 Location

### 4.1. Tissue/Fibrillar Location

MuRF1 is a muscle-specific E3 ligase, mostly present in both cardiac and skeletal muscles. MuRF1 is also present in smooth muscles where it participates to post-delivery uterine involution [96]. Skeletal muscles are mechanically and metabolically heterogenous as they contain numerous contractile and enzymatic isoforms that define different fiber types with specific contraction capacity, resistance to fatigue, energy preferences, and sensitivity to catabolic stimuli [97,98]. As MuRF1 possesses close isoforms, it is reasonable to ask whether this E3 ligase exhibits some fiber type preference. MuRF1 has been described as a fiber type II-associated factor in mice as the tibialis anterior muscle was more dependent on MuRF1 presence for developing denervation atrophy than the slow type I-containing fibers soleus [99]. However, the authors found in the same article that type I and type II fibers were similarly affected in the soleus muscle, which suggests a muscle-dependent but fiber type-independent differential expression of MuRF1. Recent work showed that MuRF1-KO protected both the tibialis anterior and soleus muscles from monocrotaline injection, indicating a similar importance of MuRF1 for both muscles regarding mass, fiber cross sectional areas, and force depletion [35]. In addition, numerous studies described an over-expression of MuRF1 in soleus muscle upon various atrophy situations [35,100,101,102,103].

### 4.2. Subcellular Location

MuRF1 is located in different subcellular compartments, i.e., linked to the myofibrillar structure, free in the cytoplasm and in the nuclei. The localization of MuRF1 seems to be partly regulated by different domains of MuRF1 for proper addressing in the different cellular compartments (Table 1).

Pioneering works focused on cardiac tissue and cultured cardiac myocytes and soon showed that MuRF1 was present in the heart in different specific location: (i) Linked to repeats A168/A169 of the giant protein titin, (ii) within the M band and the Z band (iii), within the cytoplasm [2], and (iv) also in the nucleus [104]. The interaction between titin and MuRF1 is crucial in cardiac tissues as over-expression of either MuRF1 or the titin binding site of MuRF1 disrupts the M-line region [104]. In cardiomyocytes, a model of cardiac hypertrophy induced a specific perinuclear localization of MuRF1, which was linked to a role of MuRF1 in the formation of focal adhesion through MuRF1-Rack1 interaction [107]. Still in the heart, MuRF1 was also observed linked to troponin-I, within the thin filament region of the sarcomere [28]. Altogether, this means that MuRF1 is spread in several strategical points throughout the sarcomere in the heart, in close area to its known targets (MHC, a-actin, troponin I, telethonin). Please note that, except for troponin I, all these targets were identified in skeletal muscles. Interestingly, these studies pointed out that the foci of MuRF1 vary from one myofibril to another, suggesting a dynamic localization of MuRF1 throughout the sarcomere. Another interesting point is that, in the heart, most labeling of MuRF1 was present within the sarcomere.

In skeletal muscles, few data addressed the location of MuRF1. One of the main heart locations, titin, has not been verified in skeletal muscles but the main muscle isoform of titin contains the MuRF1 binding domain in exons A168/A169 [108], suggesting a similar location in heart and skeletal muscles. Chimeric recombinant MuRF1 was mainly visualized in the cytoplasm in mice [106] while in our hands most endogenous MuRF1 is tightly linked to the myofibrils (unpublished data). This may reflect a saturation of the sarcomere binding sites by endogenous MuRF1. In human biopsies from patients suffering from different centralized nuclear myopathies, MuRF1 did not exhibit nuclear localization, unlike its close homolog MuRF2 [109]. By contrast, using an intensive care unit (ICU) rat model, both MuRF1 and MuRF2 are readily and persistently localized into the nuclei up to nine days after ICU [34]. In addition, perinuclear localization of MuRF1 and MuRF2 appeared during long-term ICU. The potential localization of MuRF1 upon catabolic stimuli is still poorly understood. For example, the nuclear accumulation observed in ICU may be due to either de novo synthesis or to the nuclear import of cytoplasmic MuRF1. In human myocytes, MuRF1 tightly associates with microtubules and co-localizes with MuRF3 [110]. In cultured myotubes from patients carrying mutations on MuRF1 and MuRF3, a disruption of microtubules occurs that can be rescued by transient expression of wild-type MuRF1 [110]. Altogether, most of the works addressing MuRF1 location were performed in heart or cardiac cells and the potential localization of MuRF1 upon muscle atrophy stimuli waits for dedicated experiments. Indeed, skeletal muscles and heart exhibit specific metabolic properties and differential location/localization of MuRF1 may occur.

## 5. MuRF1, Mutants, and Phenotypes

### 5.1. Phenotype of MuRF1-Knock-Out (KO) Mice

#### 5.1.1. Skeletal Muscle

The identification of MuRF1 as a putative universal marker of skeletal muscle atrophy prompted to genetically engineer mice with null alleles for MuRF1 gene to study its in vivo function [3]. In standard condition, MuRF1-KO mice were viable and fertile and appeared normal (normal growth curves, and skeletal muscles and heart with normal weights and morphology). The KO and wild type (WT) strains exhibited the same phenotype at birth and until month 18 [3,31], which is slightly different after month 18 (see below) [111]. However, when the gastrocnemius muscles were submitted to denervation during 14 days, a 36% sparing of muscle was observed for MuRF1-KO mice compared with WT mice [3]. These first data provided strong evidence that MuRF1 was a critical regulator of skeletal muscle atrophy.

A decade later, Labeit et al. [112] studied MuRF1-KO mice submitted to hindlimb suspension model as catabolic model. This model induces skeletal muscle atrophy but, contrary to denervation, keeps innervation intact and thus is not associated with inflammatory and stress-related pathways. After 10 days of suspension, the soleus muscle in the MuRF1-KO mice exhibited nearly complete protection against atrophy. This total protection highlights the critical role of MuRF1 during skeletal muscle atrophy, even though other E3 ligases expressed are also involved in muscle atrophy, such as MAFbx [3], MUSA1/Fbxo30 [113], TRAF6 [114], Nedd4-1 [115], SMART/Fbxo21 [61], or TRIP12 [116].

The phenotype of the MuRF1-KO mice was further studied in response to glucocorticoid treatment [117,118]. Synthetic glucocorticoids, often used to treat inflammation, induce skeletal muscle loss when used chronically or at high doses. Muscles are spared in MuRF1-KO mice following Dex treatment in parallel with the preservation of fiber cross-sectional area (CSA) [118]. Surprisingly, muscle sparing also correlated with a sustained protein synthesis rate of the triceps surae [118], by contrast with the decreased synthesis rate observed in mice following acute glucocorticoid treatments [119]. Higher protein synthesis rates were also observed in the quadriceps of MuRF1-KO mice compared to WT mice, after four days of amino acid deprivation (another catabolic situation) [29]. In the later study, MuRF1 inactivation was also reported to protect from muscle atrophy induced by amino acid deprivation.

In the different catabolic models used so far (denervation, disuse, glucocorticoid, amino acid deprivation, acute lung injury) known to lead to skeletal muscle loss, the MuRF1-KO mice present the fascinating capacity to preserve their muscle mass with no observable alteration of muscle structure [3,29,31,70,112,118]. These studies demonstrated that MuRF1 is a critical regulator of skeletal muscle atrophy during catabolic situation. However, MuRF1 seems to be non-essential to the metabolism of skeletal muscle in basal condition. This raises several questions: How are contractile proteins renewed in this mutant? Is their turnover modified? Do altered proteins accumulate? Is this apparent “non-essentiality” in basal condition due to functional redundancy with other E3s (like other MuRFs)? Or is there a compensation phenomenon due to the KO?

MuRF1 was also reported to have a role in aging. Difference between WT and MuRF1-KO mice, in basal condition, appeared at month 24. At this age, WT mice displayed muscle atrophy (decrease in muscle mass, fiber area, and maximum isometric tension), which was not the case in MuRF1-KO mice [111]. Moreover, deletion of MuRF1 also delayed the establishment of anabolic resistance in older mice (18 months). Indeed, 14 days of overload still stimulated the growth of the plantaris muscle in old MuRF1-KO mice similarly to young mice, while old WT mice lost this capacity [111].

#### 5.1.2. Cardiac Muscle Phenotype of MuRF1-KO Mice

Cardiac hypertrophy is a major risk factor for heart failure and is characterized by increased mass of cardiac muscle due to an increased protein synthesis leading to increased protein content and cell size. Since MuRF1 is also expressed in the heart, the cardiac function of this gene was also investigated. First, in cellulo studies revealed an inhibitory activity of MuRF1 on cardiomyocyte hypertrophy induced by α1-adrenergic receptor [107,120]. In MuRF1-KO mice, in basal condition, no structural or functional deficit was observed in the heart compared with control WT mice for at least up to six months of age. In vivo, cardiac hypertrophy can be induced by pressure overload, as observed during chronic hypertension, using transaortic constriction model (TAC) [121]. Upon TAC, compared with WT mice, MuRF1-KO mice exhibited an exaggerated cardiac hypertrophic response with a greater increase of anterior and posterior wall thicknesses during both systole and diastole within 14 days. This was accompanied by greater left ventricular mass index, greater heart weight/body weights, and a larger CSA of cardiomyocytes compared with the WT [121]. Two weeks after TAC, the number of TdT-mediated dUTP nick-end labeling (TUNEL)-positive nuclei (apoptosis mark) was increased in both WT and MuRF1-KO mice but the increase was higher in MuRF1-KO [120]. Similarly, the cardiac fibrosis observed after TAC was more important in MuRF1-KO mice [120]. These data suggest that the absence of MuRF1 results in more severe cardiac dysfunction of TAC. Altogether, this proves that, in vivo, MuRF1 has a protective role on heart function, in conditions leading to cardiac hypertrophy, by limiting cardiac hypertrophy, which cannot be compensated by other E3 ligases.

Part of MuRF1 cardiac protective effects may be indirect through transcriptional expression modifications. Indeed, in neonatal rat ventricular myocytes (NRVM), exposure to agonists that induce a hypertrophic response phenotype (e.g., phenylephrine (PE), angiotensin-II, endothelin-1, insulin-like growth factor 1 (IGF-1)) resulted in an up-regulation of the expression of hypertrophic markers (ANP (atrial natriuretic peptide), alpha-actin, and beta-MHC mRNA). However, the increased expression of MuRF1 in NRVM blocked the transcriptional up-regulation of these markers in response to agonists [107]. Accordingly, TAC led to the up-regulation of the expression of ANP and alpha-actin in the heart, which was stronger in the heart of MuRF1-KO mice [120].

Are the mechanisms involved by MuRF1 during cardiac hypertrophy partly similar/identical to those observed during skeletal muscle atrophy? Some clues were provided by a report addressing the role of MuRF1 in different cardiac atrophying models (TAC reversal, dexamethasone-induced- and doxorubicin-induced cardiac atrophy) [122,123]. First, the effect of chronic Dex treatments was studied on cardiac wall thickness in WT and MuRF1-KO mice, subjected to daily Dex injections or Dex release from osmotic pumps dorsally implanted [122]. Both treatments in adult mice led to a reduction in cardiac mass (in contrast to humans, where Dex is associated with increased cardiac mass). WT mice receiving Dex treatment displayed significant decreases in heart anterior and posterior wall thicknesses with parallel decrease in cardiomyocyte CSA. By contrast, MuRF1-KO mice were resistant to Dex-induced cardiac atrophy, as revealed by little or no changes in anterior and posterior wall thickness [122].

In a second mice model, cardiac atrophy was induced by injecting doxorubicin, an anthracycline widely used in the treatment of numerous malignancies. In this model, doxorubicin induced a decreased body weight and a dose-dependent decrease in heart weight and contractile function in WT mice. Moreover, intraperitoneal injection of doxorubicin at 20 mg/kg, in WT mice, caused an atrophy of myocytes (decrease of CSA) accompanied by an increase in MuRF1 mRNA and protein levels suggesting that MuRF1 could be involved in this process [123]. In accordance with this hypothesis, body weight and heart mass and contractile function were not affected by doxorubicin injection in MuRF1-KO mice when compared to WT mice [123]. These data suggested that doxorubicin-induced cardiac atrophy was in part mediated by MuRF1.

A compensatory cardiac atrophying model was also studied. This atrophy is observed after the release of pressure overload and has been proposed to be beneficial since reducing heart mass in patients with pathological cardiac hypertrophy may improve patients’ outcome [122,124]. When pathological cardiac hypertrophy was induced by four weeks-TAC (pressure overload), MuRF1-KO mice displayed higher cardiac hypertrophy (around two-fold) and higher posterior wall thickness compared with WT mice. After release, both cardiac mass and wall thickness returned to baseline within four days in WT mice. By contrast, in MuRF1-KO mice, cardiac mass only decreased by 50% and remained 32.5% higher than WT hearts in the subsequent four weeks. This defect of the MuRF1-KO heart to return to basal levels after TAC was associated with a defect of cardiomyocytes CSA to decrease back to basal levels [122]. These data highlight the importance of MuRF1 in regulating cardiomyocyte mass during compensatory atrophy to return to basal condition. Moreover, in post-catabolic situation (two weeks after cardiac infarction), the expression of MuRF1 was blunted both at the mRNA and proteins levels [125].

Altogether, these results indicate that MuRF1 is an important regulator of cardiac atrophy/hypertrophy and contractile activity.

### 5.2. MuRF1 Mutation in Humans

Mutations in the MURF1/TRIM63 gene have been reported in humans. Indeed, in the quest to determine the pathogenic role of MuRF1 in human hypertrophic cardiomyopathy (HCM), Chen and collaborators sequenced the MURF1 gene in HCM patients and controls [105]. Heterozygous mutations in the MURF1/TRIM63 gene were more frequently detected in a cohort of HCM patients than in controls (4.4% vs. 1.1%). Moreover, two heterozygous mutations (two missense: p.Ala48Val and p.Ile130Met and a deletion: p.Glu247Ter) were exclusively detected in the HCM cohort patients. When expressed in mouse hearts, these mutant MuRF1 proteins led to cardiac hypertrophy [105]. The nonsense mutation changing the glutamine at position 247 to a stop codon (p.Gln247Ter), was found in a family as either a heterozygous or a homozygous mutation [110]. The individual carrying the homozygous MuRF1 null mutation was diagnosed with HCM secondary to chronic hypertension. However, this patient was not examined for potential skeletal muscle alterations [110]. Recently, a 65-year-old patient carrying the same homozygous mutation (p.Gln247Ter) was reported. This patient presented a cardiac hypertrophy and was initially investigated for a transient ischemic attack [126]. He also presented a mild skeletal myopathy with moderate fatigability of upper limb muscles, and a muscle biopsy from vastus lateralis showed mild myopathic changes [126].

Interestingly, the available data describe similar phenotypes in both patients carrying a homozygous non-sense mutation and MuRF1-KO mice. The invalidation of MuRF1 in humans mostly impacts cardiac function with no or moderate alterations on skeletal muscles as only hypertrophic cardiomyopathy or transient ischemic attack helped reveal these mutations. This resembles the absence of specific MuRF1-KO skeletal muscle phenotype observed in mice within standard conditions. Altogether, human and rodent studies on MuRF1 null function support a role of MuRF1 in the prevention of cardiac hypertrophy. More studies likely including catabolic situations will be necessary in humans to detect potential negative impacts of MuRF1 invalidation on skeletal muscles.

### 5.3. MuRF1 Dominant Negative Mutant

A MuRF1 dominant negative mutant was generated in mice, corresponding to a RING-deletion of MuRF1 (MuRF1-ΔRING) [106]. This truncated form of MuRF1 can no more interact with UBE2 enzymes, and thus binds, but cannot ubiquitylate substrates. The mutant mice were submitted to unilateral atrophy of the hindlimb muscles, induced by sciatic nerve section. Skeletal muscles from WT mice lost around 35% of their mass and only 20% in the MuRF1-KO mice as previously reported [3]. The MuRF1-ΔRING mice were protected from muscle atrophy in the same extent than the MuRF1-KO mice, indicating that the ubiquitin ligase function of MuRF1 is essential for the atrophy process [106].

### 5.4. Mutant Over-Expressing MuRF1

#### 5.4.1. Skeletal Muscle

Mice specifically over-expressing MuRF1 in skeletal muscles were generated [127]. These mice (MuRF1-TG) presented a 16-fold increase in MuRF1 protein expression. No difference was observed between the MuRF1-TG and WT mice, in standard conditions, regarding viability, fertility, body, and muscle mass, the myofibrillar ultra-structure, and the levels of ubiquitinated proteins in quadriceps muscle. A genome-wide transcriptional profiling of the MuRF1-TG quadriceps revealed a mild (7% to 31%) alteration of several genes encoding for enzymes involved in lipid metabolism and its coordination with glycolysis [127]. In the MuRF1-TG mice, plasma insulin levels were increased about three-fold, one hour after injection of glucose and remained elevated up to two hours. Moreover, glycogen stores were depleted in the liver (but not in muscle) of MuRF1-TG mice under basal conditions. The authors suggested that skeletal muscle MuRF1 over-expression profoundly affects carbohydrate metabolism in the liver and systemic glucose metabolism by stimulating pancreatic insulin secretion [127].

#### 5.4.2. Heart

Transgenic mice specifically over-expressing MuRF1 in the heart were also generated (MuRF1-Tg+) [122]. These mice presented thinner ventricular wall than WT mice, a decrease in cardiac function, and were predisposed to cardiac heart failure. Structurally, MuRF1-Tg+ cardiomyocytes displayed smaller CSA before and after TAC and a mild disruption of the M-line, that was accentuated after TAC. Ultra-structure modification was also previously reported in cultured cardiac myocytes where MuRF1 over-expression disrupted the structure of the C-terminal part of titin (MuRF1 binding region), suggesting that MuRF1 regulates the stability of this large structural protein [104] (cf § 6.2). MuRF1-Tg+ versus WT mice were analyzed by microarray, in both standard conditions and during pathological cardiac hypertrophy [122]. Most of the genes whose expression changed in MuRF1-Tg+ mice were involved in cardiac energy metabolism. Moreover, the activity of creatine kinase (CK) in these hearts was significantly depressed, but without CK protein levels modification [122]. CK is a key player in energy metabolism since it buffers cellular energy and maintains energy homeostasis [128]. This suggests that energy-linked proteins are controlled by MuRF1 in heart.

It is noteworthy that the over-expression of MuRF1 in skeletal muscle does not lead to muscle atrophy in standard condition, and over-expressing MuRF1 in heart induces heart failure instead of protecting against deleterious cardiac hypertrophy. This suggests that MuRF1 is necessary but not sufficient to lead to skeletal muscle atrophy and cardiac hypertrophy, and that other cofactors/regulators are necessary to finely tune these processes.

### 5.5. MuRF1/MuRF2 Double Knock-Out

As MuRF1-KO mice, MuRF2-KO mice have no obvious phenotype in standard condition. However, when homozygous constitutive MuRF1/MuRF2 double KO (M1/M2 dKO) were generated, 74% of homozygous dKO mice died within the first 16 days of life, putatively from heart insufficiency [31]. By contrast with MuRF single KO, M1/M2 dKO survivors developed cardiac and skeletal muscle hypertrophy in standard condition, during their life span [31]. Fibers from dKO mice were hypertrophied and cardiomyocytes presented abnormal myofibril with increased levels of electron-dense Z-disks. In dKO myocardium, an increased expression of phospho-p70S6K and its substrate phospho-S6 witnessed an over-activation of the Akt/mTOR pathway and paralleled de novo protein synthesis. This hypertrophy may also result from hyperactive anabolic stretch-sensing pathways. Indeed, the authors monitored protein markers for stretch signaling such as ANP (in the heart) and MLP/Csrp3 and telethonin (in skeletal muscle) and found that they were elevated in both basal and immobilized conditions. They suggested then that combined inactivation of MuRF1 and MuRF2 leads to chronic up-regulation of stretch signals and/or their deficient down-regulation.

It is noteworthy that dKO mice accumulated less body fat than WT mice. This could also be explained by hyperactive anabolic stretch-sensing pathways, a larger fraction of dietary calories being recruited by the activation of muscle protein synthesis.

### 5.6. MuRF1/MuRF3 Double Knock-Out

MuRF3-KO mice do not exhibit noticeable phenotype in standard condition but are predisposed to cardiac rupture after myocardial infarction [129]. Conversely, still in standard condition, MuRF1/MuRF3 double KO (M1/M3 dKO) mice undergo skeletal muscle myopathy and hypertrophic cardiomyopathy associated with less mobility, and walking and climbing impairment compared with WT and MuRF single mutants [129]. Moreover, M1/M3 dKO mice display a significant lower body weight than WT and single mutants. In addition, the dKO mice present an abnormal sub-sarcolemmal accumulation of myosin heavy chain (MHC) fragments in skeletal and cardiac muscles, a phenotype observed in all fiber types. This was attributed to the capacity of MuRF1 and MuRF3 to target MHCIIa and β/slow MHC for UPS-dependent degradation [129].

A similar phenotype was observed in humans for a patient suffering from hypertrophic cardiomyopathy (HCM) and skeletal myopathy [110]. His muscle fibers contained inclusions of myosin and myosin-associated proteins. The patient was homozygous for a null mutation in MURF1 gene and heterozygous for a MURF3 missense mutation. Genetic analysis confirmed that other members of the family carried these mutations but only the homozygous MuRF1 null mutation combined with the MURF3 heterozygous mutation led to HCM and skeletal myopathy [110].

Altogether, the absence of a single MuRF does not have any adverse effect, but the invalidation of two members of the family ends up with pathological phenotypes. The simplest hypothesis is that the MuRFs exhibit at least partial redundancy of function/targets in mammals, which implicates some constraints for the development of future pharmacological inhibitors, e.g., for fighting against muscle atrophy. Indeed, these inhibitors will have to target a single MuRF isoform and/or a specific set of substrates while leaving intact the other functions.

## 6. Interacting Partners

Since MuRF1 is a key player in the development of skeletal muscle atrophy and in the protection against cardiac hypertrophy, many studies have focused on the identification of MuRF1 partners to better understand its mode of action. Numerous proteins have been identified as potential partners mainly from yeast two-hybrid (Y2H) screens performed on dedicated library (heart, skeletal muscle, or cytoplasmic proteins), some of them being confirmed and others waiting for more studies. These partners are either MuRF1 substrates (e.g., a-actin, MHC, etc.), MuRF1 interactors without being a substrate (e.g., titin, Bif-1/EndoB1, etc.), or are influenced by MuRF1 in their metabolic functions (directly or not; e.g., PPARa, etc.). This chapter will depict what is known on these MuRF1-interacting proteins and the metabolic pathways that may be influenced by MuRF1.

### 6.1. MuRF1 Substrates

Although, the list may be incomplete, studies have already identified several MuRF1 substrates. To be defined as substrates, proteins have to fulfill different criteria: To directly interact with MuRF1; to be stabilized by the presence of proteasome inhibitor; to be destabilized in an in cellulo degradation/stability assay by MuRF1, or to accumulate in absence of MuRF1.

#### 6.1.1. In Skeletal Muscle

##### Myofibrillar/Contractile Proteins

**Figure 3:**
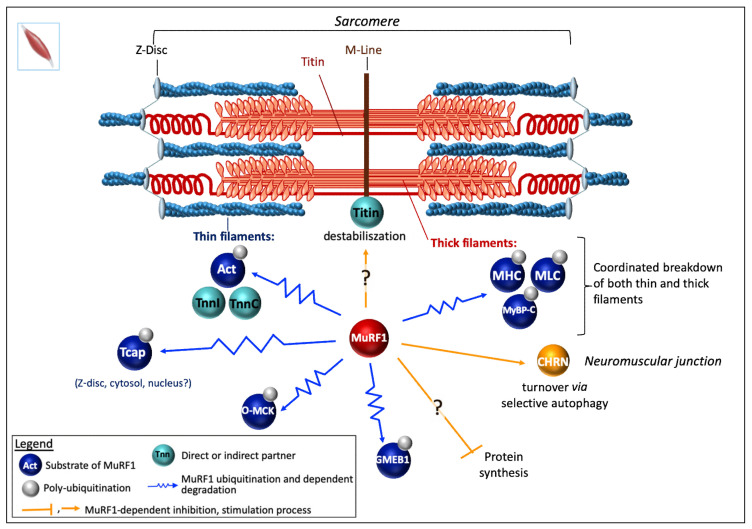
MuRF1 functions in skeletal muscle. MuRF1 is responsible for the coordinated breakdown of both thick and thin filament occurring during catabolic states in skeletal muscle. Like in cardiac muscle, MuRF1 probably binds to titin at the M-line in some conditions (catabolic conditions, MuRF1 excess, etc.), causing the disruption of titin/M-line interactions and hence a partial disorganization of sarcomere assembly. Upon sarcomere destabilization, MuRF1 would have access to contractile proteins (alpha-actin, troponins I, C, and T, MHC, MLC, MyBP-C), to target them for polyUb and proteasome-dependent degradation. MuRF1 also drives telethonin (Tcap) degradation, but it remains to determine which form of the telethonin is targeted by MuRF1: The free form, the titin-complexed form, or both. The inhibition of protein synthesis by MuRF1 has been reported in skeletal muscles, but more investigations are needed to confirm this role. MuRF1 was reported to target the non-functional oxidized form of muscle creatine kinase (O-MCK). CHRN is targeted to the LC3-II-positive autophagosome for selective autophagy under catabolic condition. This process is regulated and/or mediated partly by MuRF1 and its interacting partners Bif-1/EndoB1 and SQSTM1/p62. Act, alpha-actin; CHRN, cholinergic receptor, nicotinic/nicotinic acetylcholine receptor; GMEB1, glucocorticoid modulatory element binding protein-1; MHC, myosin heavy chain; MLC, myosin light chain; MyBP-C, myosin binding prot-C; O-MCK, oxidized form of muscle creatine kinase; TnnI, TnnC, troponin I and C.

Enhanced myosin heavy chains (MHC) degradation had been attributed, in a pioneering work, to the UPS in skeletal muscles from both ground-based unweighted and spaceflight rats, but the mechanism was not reported [130,131]. Thereafter, two laboratories concomitantly reported that MuRF1 targets MHCIIa and ß/slow MHC for UPS-dependent degradation, leading to the discovery of the first MuRF1 substrate [117,129]. Clarke and collaborators showed that Dex treatment induced a loss of MHC in myotubes that can be blunted by inhibiting the proteasome [117]. This loss of MHC was also attenuated in MuRF1-KO mice and both studies demonstrated that MuRF1 and MHC interact. The authors also checked that the decrease of MHC proteins was not due to a decrease in their mRNA levels in catabolic conditions. Altogether the results suggested that MuRF1 targets MHCIIa and ß/slow MHC to UPS-dependent degradation (Figure 3).

**Myosin binding prot-C** (**MyBP-C**) **and the light chain of myosin** (**MLC**). Complementary works supported that MyBP-C and MLC were also targets of MuRF1 [21,105,106,132] (Figure 3). Direct interaction was demonstrated by Y2H approach using a skeletal muscle library for MLC [21] and an adult heart cDNA library for MyBP-C [132]. A parallel between atrophying muscles, increase in MuRF1, and a loss of MLC and MyBP-C was observed upon denervation or fasting conditions [106]. However, most analyses have been made using SDS-PAGE and Coomassie blue staining. MLC1, MLC2, and MyBP-C were further shown to be ubiquitinated by MuRF1 using an in vitro assay but with non-cognate E2s for MuRF1 (see §6.3) [106].

**Alpha-actin** (a-actin) is the major protein present in skeletal muscle, hence the importance of deciphering how it is controlled during atrophy situations. Intriguingly, some authors reported that a-actin and MHC degradation were disconnected as a-actin remained stable for days while MHC was either degraded in a MuRF1-dependent mechanism in atrophying muscles or accumulated in MuRF1-KO mice [117,129]. Another study mostly based on Coomassie blue assays also concluded to a delayed degradation of a-actin when compared to MHC upon catabolic stimuli and that MuRF1 was not implicated in a-actin degradation [106]. These data are in contradiction with other reports that showed that in humans a-actin/MHC ratio is stable upon bedrest and microgravity combined or not with exercise [133,134]. The disconnection between MHC and a-actin degradation was also challenged by Cosper and Leinwand, who showed that wrong experimental design led to erroneous conclusions [135] (compare [106,117,129] with [135]). Indeed, the use of an inappropriate buffer ends up with poor solubilization of MHC and results in artifactual data [135]. Cosper and Leinwand clearly demonstrated that MHC and a-actin are coordinately degraded in catabolic muscles [135] (Figure 3). Furthermore, using several complementary approaches, other works finally showed that a-actin is clearly a UPS substrate rapidly degraded upon catabolic stimuli and that MuRF1 is the major E3 ligase involved [136,137] (Figure 3). Firstly, polyUb a-actin was detected in vivo in both C2C12 myotubes subjected to Dex treatment and in human biopsies from cancer patients [136]. Secondly, sarcomeric flag-tagged actin degradation was blunted only by using the proteasome inhibitor MG132. Thirdly, MuRF1 knockdown using siRNA approach completely abolished a-actin degradation in catabolic C2C12 myotubes. Finally, MuRF1 was also shown to directly interact (in vitro and ex-vivo) with a-actin and to further induce its polyubiquitination [136].

Altogether, MuRF1 is the main driver of MHC and a-actin, the two main components of myofibrils. In addition, MuRF1 is also able to interact with the skeletal muscle isoforms of troponin I and T [21], which suggests that MuRF1 may be a central regulator for coordinating the breakdown of both thin and thick filaments making MuRF1 a highly interesting target for developing therapeutic approaches.

**Telethonin.** Recently, another structural protein, namely telethonin, has been described as a new MuRF1 target [138] (Figure 3). Telethonin, also known as titin-cap protein or Tcap, participates with titin in anchoring filaments to the Z-disc of the sarcomere. Thereby telethonin gene mutations lead to muscular dystrophy LGMD2G (limb-girdle muscular dystrophy type 2G) [139] and cardiomyopathies [140,141]. Telethonin turned out to be a multifunctional protein present in the Z-line (complexed with titin), free in cytoplasm or in the nuclei, potential role being also suggested [142,143,144,145,146]. Telethonin was identified as a 26S proteasome substrate in atrophying rat muscles, using a polyUb affinity matrix [137,147]. Telethonin has been shown to interact with MuRF1 using a Y2H system and also in mammalian cells, using a split-GFP approach [21,138]. Recently, MuRF1 was reported to drive telethonin degradation in an in cellulo degradation assay [138]. However, it remains to determine which form of the telethonin is targeted by MuRF1: The free form, the titin-complexed form, or both.

##### Miscellaneous

**Muscle-type creatine kinase (MCK**) has been proposed to be a target of MuRF1. MCK binds to the M-line of sarcomere and functions as an ATP supplier combined to actin-activated myosin ATPase [148,149]. MCK was initially found to directly interact with MuRF1 in a Y2H screen [21]. This was further confirmed by GST-pulldown experiments performed using COS7 cells co-expressing GST-MuRF1 and Flag-MCK, the interaction being mediated by the B-box of MuRF1 (Figure 1) [29]. In these cells, co-expression of MuRF1 and MCK also led to a mild MCK ubiquitination only observable in the presence of MG132. Similar data were obtained with MuRF2 and MuRF3 [29]. The invalidation of MuRF1 in mice was reported to correlate with an increased activity of creatine kinase (CK) and, conversely, the over-expression of MuRF1 in mice correlated with a decreased CK activity [29,122,148,149,150]. However, several isoforms of CK (including MCK) co-exist in cells and it is also known that modulation of CK activity can be achieved through phosphorylation [150,151,152] or oxidation [153]. Oxidation of MCK (O-MCK) impedes its binding to the M-line protein myomesin and it was also reported that O-MCK is more prone to MuRF1-dependent ubiquitination in vitro assay [153]. Altogether, whether MuRF1-dependent targeting of MCK occurs in skeletal muscles remains an open question.

**Glucocorticoid modulatory element binding protein-1 (GMEB1) and 3-hydroxyisobutyrate dehydrogenase (HIBADH**) have been proposed to be MuRF1 substrates. Both GMEB1 and HIBADH interact with MuRF1 using Y2H screen approaches [21,104] and they are ubiquitinated by MuRF1 when co-expressed with MuRF1 in COS7 cells [29]. GMEB1 is a nuclear transcriptional regulator that binds to glucocorticoid modulatory elements (GME) in response to changes in cellular glucocorticoid levels. GMEB1 is also present in the cytosol but the cytosolic functions have been poorly studied and may be related to pro-caspase inhibition [154]. HIBADH is one of the key enzymes involved in valine degradation [155]. An inhibition of HIBADH by MuRF1 would suppress branched chain amino acids (BCAA) consumption as energy source or for biosynthetic aims. However, in mammals, HIBADH is localized in mitochondria [155], and up to now MuRF1 has not been unambiguously localized in mitochondria, which raises questions about a putative role of MuRF1.

#### 6.1.2. In the Heart

##### Myofibrillar Proteins 

**Figure 4:**
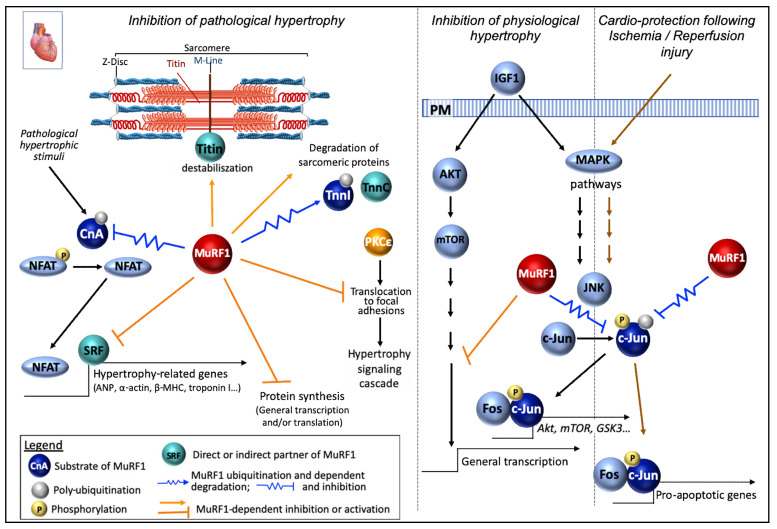
MuRF1 functions in cardiac muscle. MuRF1 exhibits a protective role in the heart by inhibiting pathological heart hypertrophy. Anti-hypertrophic function of MuRF1 can partly be explained by its atrophic function and the degradation of sarcomeric proteins like in skeletal muscle. Moreover, MuRF1 inhibits the protein synthesis of hypertrophy-related genes in response to hypertrophic stimuli, via the inhibition of at least three pathways (serum response factor (SRF), c-Jun N-terminal kinases (JNK), and AKT pathways). The activation of calcineurin/NFAT (nuclear factor of activated T cells) signaling alone is sufficient to induce pathological cardiac hypertrophy. MuRF1, by down-regulating the phosphatase CnA, prevents the translocation of the nuclear factor NFAT transcription from the cytosol to the nucleus and thus the consecutive transcription of hypertrophy-related genes. Moreover, MuRF1 mediates the inhibition of PKCε translocation to focal adhesions, a critical event in the hypertrophic signaling cascade. MuRF1 also limits physiological cardiac hypertrophy by inhibiting c-Jun protein expression and phosphorylation in the presence of insulin-like growth factor 1 (IGF-1). Another cardioprotective role of MuRF1 is to reduce cardiomyocytes apoptosis occurring after ischemia/reperfusion injury (I/R). During I/R, MuRF1 targets phospho-c-Jun for degradation, blocking the transcription of pro-apoptotic genes; it is noteworthy that the inhibition of JNK or c-Jun is cardioprotective in vivo. Act, alpha-actin; CnA, calcineurin A; IGF-1, insulin-like growth factor 1; MHC, myosin heavy chain; MLC, myosin light chain; MyBP-C, myosin binding prot-C; NFAT, nuclear factor of activated T cells; SRF, transcription factor serum response factor; TnnI, TnnC, troponin I and C.

**Troponin I** was detected as a MuRF1 partner in a Y2H screen using a human heart library [28]. The interaction was confirmed by immunoprecipitation and GST-pulldown from neonatal rat ventricular myocytes (NRVM) previously infected either with Ad.GFP-Myc-MuRF1 or GST-Myc-MuRF1, respectively. The interaction domain was mapped to the first coiled-coil region located between amino acids 153 and 188 in MuRF1 (Figure 1). Increasing levels of MuRF1 destabilize troponin I in a cell degradation assay, performed using COS-7 cells co-transfected with plasmids expressing MuRF1 and troponin I [28]. Similar data were obtained analyzing the endogenous levels of troponin I in NRVM infected with Ad.GFP-Myc-MuRF1 at increasing titers. Moreover, ubiquitination levels of troponin I increased in NRVM infected with Ad.GFP-Myc-MuRF1. Pulse–chase analysis of [35S] methionine plus cysteine-labeled NRVM confirmed that MuRF1 reduced the half-life of endogenous troponin I from 17 h to 4 h after adenoviral expression of MuRF1 [28] (Figure 4). Accordingly, troponin I accumulates in the heart of MuRF1-KO mice [120].

**Myosin heavy chain (MHC)**. Cardiac myocytes transduced with Ad-TRIM63Q247* (a MuRF1-deletion variant found in HCM patients) displayed almost no ubiquitination on MYH6 [105]. It should be noted that this mutant behaves as a dominant negative mutant. Accordingly, MYH6 expression was raised in cardiomyocytes transfected with Ad-TRIM63Q247* compared with cells transfected with the WT form, without any change in mRNA levels [105], suggesting that MuRF1 targets MYHC in both skeletal and cardiac muscle (Figure 4).

**MyBP-C.** Studies on the heart confirmed the data obtained in skeletal muscle for MyBP-C (Figure 4). Indeed, in MuRF1-KO mice, the absence of MuRF1 was associated with higher cMyBP-C (cardiac MyBP-C isoform) protein levels. Accordingly, in MuRF1-TG mice, the over-expression of MuRF1 was associated with lower cMyBP-C protein levels [132]. Accordingly, cardiomyocytes, transduced with the human dead MuRF1 mutant (TRIM63Q247*), displayed a near complete absence of ubiquitinated MyBP-C compared to myocytes transfected with WT MuRF1 [105]. Moreover, MyBP-C protein levels were reduced in cardiomyocytes over-expressing the WT form of MuRF1, compared to non-transduced cells, and conversely, were increased in cells transfected with the dead form. These changes were not due to altered mRNA levels [105].

##### Signaling and Protein Synthesis

**Calcineurin A.** Calcineurin A (CnA) is a calcium/calmodulin-dependent serine/threonine phosphatase which is up-regulated in the heart in response to hypertrophic stimuli. CnA activity is regulated at the protein level [156] and by endogenous inhibitors (for ex MCIP1, CIB1) [157,158]. Upon dephosphorylation of the nuclear factor of activated T cells (NFAT) family, CnA induces NFAT translocation from the cytosol to the nucleus and the consecutive transcription of hypertrophy-related genes such as ANP, a-actin, and ß-MHC [159]. Activation of calcineurin/NFAT signaling alone is sufficient to induce pathological cardiac hypertrophy [160].

MuRF1 has been involved in the negative regulation of CnA protein [120]. Indeed, the increase in CnA activity observed after TAC was enhanced in MuRF1-KO mice. Moreover, the down regulation of MuRF1 (sh-RNA) lead to an increase in CnA stability in cardiomyocytes. Accordingly, polyUb CnA levels upon TAC was reduced in the heart of MuRF1-KO mice. Finally, both MuRF1 and Cna mutually co-immunoprecipitate one another [120]. These results were in agreement with a previous report indicating that myocytes transduced with the human WT form of MuRF1 displayed an increase in the ubiquitination of CnA [120]. By contrast, polyUb CnA levels were drastically reduced in cardiac myocytes transduced with the human missense mutation TRIM63Q247* form of MuRF1, without any change in its mRNA levels [105]. MuRF1 may thus modulate cardiac hypertrophy by down-regulating CnA-NAFT signaling pathway in parallel with the targeting of contractile proteins in a coordinate way (Figure 4).

**Phospho c-Jun.** In cardiomyocytes, MuRF1 over-expression protects from an ischemia/reperfusion (I/R) by decreasing cell death and apoptosis [22]. Myocardial ischemia occurs when factors occlude the coronary arteries. Therapeutic interventions allow heart reperfusion and limit the size of the infarct, but also have detrimental issues such as inflammation and the development of apoptosis. Different MAPK signaling pathways are involved in I/R, especially the c-Jun N-terminal kinases (JNK) pathway that is activated, the inhibition of JNK or c-Jun being cardioprotective in vivo [161,162]. JNK phosphorylates c-Jun that can then bind to fos proteins to constitute AP-1 transcription factors, one function being to induce apoptosis in response to I/R injury (Figure 4). Increasing the expression of MuRF1 decreased the level of phospho-c-Jun (S63) in cardiomyocytes challenged by I/R, which was not the case in normoxic conditions. Similar data were obtained using MuRF1-Tg+ mice (specifically over-expressing MuRF1 in the heart) submitted to I/R [22]. The other MAP kinases pathways were not affected by the levels of MuRF1. Conversely, decreasing MuRF1 levels resulted in an increase in phospho-c-Jun during I/R concomitant to an enhanced activation of AP-1 activity in cardiomyocytes. MuRF1-Tg+ mice were protected against I/R injury with a maintained heart function, decreased heart dilatation, and smaller infarction after injury compared to WT mice. MuRF1 action seems direct as it interacts with c-Jun and exhibits a better affinity for the phosphorylated form (Figure 1). In addition, MuRF1 is able to ubiquitinate phospho-c-Jun in an in cellulo degradation assay [22].

c-Jun/MuRF1 interconnection was also described during physiological cardiac hypertrophy (for instance occurring in an athlete’s heart in response to repetitive exercise), which is developed in response to insulin-like growth factor (IGF-1) via the activation of IGF-1-Akt signaling [163]. In cardiac-derived cells (HL1) and primary cardiomyocytes (NRMV) challenged with IGF-1 stimulation, MuRF1 knock-down results in an enhanced cell growth, while MuRF1 over-expression inhibits IGF-I cardiomyocyte hypertrophy [164]. Moreover, the IGF-1-induced increase of Phospho-GSK3b (an immediate downstream effector of Akt) was blunted by increased MuRF1 expression in cardiac-derived cells and primary cardiomyocytes. Here again, MuRF1 was able to inhibit c-Jun protein expression and phosphorylation in the presence of IGF-I. Moreover, JNK inhibitor SP-600125 was able to prevent the cardiomyocyte hypertrophy stimulated by IGF-I observed when MuRF1 was knocked-down [164]. Furthermore, using MuRF1-KO-, MuRF1-Tg+ and WT mice submitted to five weeks of voluntary running, the authors conclude that MuRF1 inhibits exercise-induced cardiac hypertrophy in vivo [164] (Figure 4). This is consistent with a previous study reporting a general translational activation in MuRF1/MuRF2 double KO (but not in the single KO) mice myocardium via the activation of the AKT/mTOR pathway (upregulation of p70S6K and its substrate phospho-S6) [31]. Interestingly, the MuRF1-KO mice ran farther than the WT and MuRF1-Tg+ mice [164]. The current hypothesis is that the control of cardiac hypertrophy by MuRF1 may at least partly be due to the inhibition of JNK/AP-1 signaling through MuRF1-dependent targeting of phospho-c-Jun.

**The transcription factor serum response factor (SRF).** MuRF1 has been shown to interact with the transcription factor SRF (an inducer of pro-hypertrophic gene expression) and to inhibit its activity in COS7 cells [121]. Accordingly, the expression of SRF-dependent genes (beta-MHC, smooth muscle actin, BNP) was enhanced in MuRF1-KO mice submitted to TAC hypertrophy model [121] (Figure 4). It should be noted that the MuRF1-SRF interaction reported in COS7 cells, may be indirect since no direct interaction can be detected between MuRF1 (or MuRF2) and SRF, using Y2H approach [31]. It is noteworthy that in basal condition, M1/M2 dKO mice did not exhibit SRF modulation (mRNA, proteins levels, nuclear recruitment) in the myocardium [31]. However, an increased expression of markers of cardiac hypertrophy was observed in dKO hearts. These transcriptional changes resembled those present in pressure-induced aortic constriction [165], including the upregulation of skeletal-type alpha actin 1, myosin light chains, atrial natriuretic peptide (ANP). Other work reported that, in cardiomyocytes and skeletal muscle, mechanical inactivity induced MuRF2 translocation in the nucleus and subsequent binding to nuclear SRF, which reduces SRF transcriptional activity [109]. Whether MuRF1 uses a similar mechanism to control SRF remains to be determined, but by using KO animals it was shown that MuRF1 is essential for normal cardiac response to mechanical stress (TAC) while MuRF2 is dispensable [121].

### 6.2. Other MuRF1 Interactors (Not or Not Yet Substrates)

**Bif-1/EndoB1/SH3GLB1 and SQTM1/p62.** Bif-1 (Bax interacting factor 1)/EndoB1 (Endophilin B1) and SQTM1/p62 (Sequestosome-1) have been identified as interacting partners of MuRF1 in a Y2H screen [31] (Witt 2008). Interaction with Bif-1 was further confirmed by MuRF1 immunoprecipitation on quadriceps muscle lysates [166]. Endophilins are peripheral membrane proteins involved in sensing and inducing membrane curvature during vesicle formation [167]. Bif-1/EndoB1 has been linked to intracellular trafficking pathways and the larger part of Bif-1 is associated with small intracellular vesicles [168]. More specifically, in mouse tibialis anterior muscle, a small proportion of the protein is observed in a striated pattern of the sarcomeric region while the majority is located near the neuromuscular junction (NMJ) region [166]. Bif-1 has also been shown to localize to autophagosomes with Atg5 and microtubule-associated protein light chain 3 (MAP1 LC3), and loss of Bif-1 suppresses autophagosome formation [169].

MuRF1 co-localizes and is highly enriched with Bif-1 at the NMJ. MuRF1 also co-precipitates with acetylcholine receptor (CHRN or cholinergic receptor, nicotinic/nicotinic acetylcholine receptor), the major postsynaptic ion channel of the NMJ [166]. The authors showed that under standard conditions MuRF1 did not affect CHRN stability. Indeed, both wild-type and MuRF1-KO muscles exhibited completely normal NMJ morphology and apparent CHRN turnover. However, atrophic conditions (denervation, starvation) led to a decrease in the half-life of CHRN and an enhanced internalization of synaptic CHRN suggesting an increased turnover [166,170]. Indeed, this transmembrane ion channel has to be endocytosed in vesicular carriers to get degraded in lysosomes. In MuRF1 KO mice, this process was less pronounced [166,170], leading the authors to hypothesize that MuRF1 may link sarcomeric and CHRN remodeling (Figure 3).

In vivo imaging study of mouse muscle, from the same laboratory, revealed that starvation and denervation induced a significant increase of the endocytic vesicles containing CHRN per NMJ. These vesicles were also largely positive for MuRF1 and for some autophagy/lysosomal markers including LC3, SQSTM1/p62 (another interactor of MuRF1), and LAMP1 [170]. Noteworthy, LC3 labelling did not overlay but surrounded these puncta. In MuRF1-KO mice muscles, the co-localization rate between Bif-1 and CHRN as well as between LC3 and CHRN was significantly lower in control conditions.

On the whole, CHRN is targeted to LC3-II-positive autophagosome for selective autophagy under catabolic condition and this process is regulated and/or mediated by MuRF1 and its interacting partners Bif-1/EndoB1 and SQSTM1/p62 [170] (Figure 3). Further work should determine the mechanism involving MuRF1 in selective autophagy and whether MuRF1 could similarly be implicated in the selective autophagy of other membrane proteins. One can hypothesize that this process involves the degradation of Bif-1/EndoB1 and/or SQTM1/p62. A first line of evidence comes from M1/M2-dKO mice heart where SQSTM1/p62 protein levels are strongly increased while the mRNA content is not modified which suggests post-transcriptional regulation [31].

**DDB1 and cullin-associated factor 8 (DCAF8).** DCAF8 (WD repeat containing protein 42A, WDR42A) is a substrate receptor for cullin RING ligases (CRLs) belonging to the CRL4 complexes [171]. MuRF1 interacts with DCAF8 and immuno-precipitate with DCAF8 and DDB1, Cul4A, RBX1, that are members of CRL4 complexes [172]. The authors proposed that DCAF8 and MuRF1 may act synergistically to degrade MHC. However, a DCAF8-linked degradation of MuRF1 cannot be ruled out from these experiments. Indeed, the authors’ conclusion relies on C2C12 experiments treated with pharmacological doses of Dex (100 X the concentration routinely used), which might have biased the data. In addition, they used a non-adequate buffer to extract contractile proteins (see § 5.1.a and [135]). In conclusion, more work is still needed to define the functional meaning of the DCAF8-MuRF1 interaction.

**PPARα.** Peroxisome proliferator activated receptors (PPARs) are a subfamily of nuclear hormone receptors that form heterodimers with retinoid X receptors and regulate the transcription of several genes involved in lipid metabolism, energy utilization, and storage [173]. There are three isoforms of PPARs (PPARα, PPARβ/δ, and PPARγ). Using MuRF1 over-expression and knock-down in NMRV and HL-1 cardiomyocytes, MuRF1 was shown to inhibit cardiac fatty acid (oleate) oxidation and enhance glucose oxidation, however with no net change in ATP production [174]. In an attempt to explain the effect of MuRF1 on fatty acid oxidation observed in cardiomyocytes, Rodriguez and collaborators focused on PPAR transcription factors. They reported an array of presumptions for nuclear MuRF1 having a role on PPARα activity and localization [174]. In both COS7 and cardiac-derived HL-1 cells, the co-transfection of a plasmid containing a luciferase reporter driven by a PPAR response element (PPRE) and PPARα resulted in enhanced PPAR activity. Conversely, the co-expression of MuRF1 in this system significantly inhibited PPAR activity, while having no effect on PPARß/δ nor PPARγ activity. Moreover, increasing MuRF1 expression in HL-1 cells resulted in a decrease of the nuclear localization of PPARα in favor of cytoplasmic localization. This effect was blocked when cells were treated with Leptomycin B, an inhibitor of exportin-mediated nuclear export [174]. However, and even though MuRF1 and PPARα co-precipitated in cultured cells, whether this was a direct or an indirect effect of MuRF1 remains to be established.

The authors found also a moderate multi mono-Ub of PPARα by MuRF1 in vitro, but a highly promiscuous E2 (E2D3) was used and we demonstrated that such an in vitro assay should be cautiously interpreted ([38] and §5.5). It is also noteworthy that these effects of MuRF1 on PPARα activity and localization were not consistent with the PPARα-regulated gene expression [174]. This was explained by the authors by the multiple roles of MuRF1 in cardiomyocyte and the multiple levels of regulation of PPARα.

**RACK1 (receptor of activated protein C kinase 1) and PKCε.** RACK1 is a multifunctional scaffolding protein involved in the recruitment, assembly, and/or regulation of a variety of signaling molecules. RACK1 plays a role in several cellular processes like nucleating cell signaling hubs, anchoring proteins at specific subcellular locations and regulating protein activity [175]. RACK1 was identified as a MuRF1 partner using a Y2H screen of a human heart cDNA library [107]. Co-immunoprecipitation experiments in COS7 cells overexpressing RACK1 and MuRF1 confirmed this interaction [107]. RACK1 associates with PKCε in hypertrophied heart lysates [176] and the presence of PKCε was confirmed in RACK1/MuRF1 immunoprecipitates [107], suggesting that MuRF1 formed a ternary complex with RACK1 and PKCε isoforms. PKCε is involved in cardiac hypertrophy due to its translocation to focal adhesions, which is a critical event in the hypertrophic signaling cascade [177]. The interaction between MuRF1 and RACK1 and PKCε seemed to regulate the localization of these two proteins, in response to hypertrophic agonists (alpha-adrenergic receptor agonists) such as PE. Indeed, the presence of these agonists on NMRV cardiomyocytes resulted in co-localization of MuRF1 and RACK1 from the cytosol and sarcomere to the perinuclear region and nucleus [107]. However, the observed translocation of PKCε from perinuclear structures to focal adhesions upon activation with phenylephrine in NRVM was inhibited by the presence of MuRF1. Moreover, in Ad.MURF1-infected cells, after PE or PMA treatments, PKCε activity was more abundant in the soluble fraction and not in the fraction containing focal adhesions elements. These data support an inhibition of PKCε translocation to focal adhesions mediated by MuRF1 [107] (Figure 4). However, it remains to be determined whether these effects are dependent on the capacity of MuRF1 to interact with RACK1.

**SUMO-conjugating enzyme UBC9/UBE2I.** MuRF1 directly interacts with the SUMO-conjugating enzyme UBC9, also named UBE2I [104]. This enzyme may be responsible for the sumoylation of MuRF1 (please refer to the § 2.2 for further details on SUMO-modified MuRF1).

**Titin.** Titin, also known as connectin, is the largest vertebrate protein (~3800 KDa). Titin molecules compose the third filament system in the striated muscle sarcomeres, besides the thick and thin filaments. Each titin molecule extends to half sarcomeres from Z-disks to M-bands. Titin’s N-terminal (~80 KDa) region is integrated in the sarcomeric Z-disks, and its C-terminal (~200 KDa) region is located in the M-band [178]. The majority of the protein is composed of tandem repeats of immunoglobulin (Ig)-like and fibronectin (FN3)-like domains. This protein possesses a molecular spring (in the I-band) that provides elasticity and allows post-contraction recovery as well as a kinase domain for signaling, titin being recognized as a stretch sensor [179].

MuRF1 interacts with the titin repeats A168/A169 adjacent to the C-terminally located titin kinase domain [2] (Figure 1). Over-expression of MuRF1 in cardiac myocytes disrupts the structure of the C-terminal part of titin, including the MuRF1 binding domain, suggesting that MuRF1 regulates the stability of this large structural protein [104]. Of note, no ubiquitination-linked degradation of titin was attributed to MuRF1. Around 65% of the cardiomyocytes over-expressing MuRF1 also displayed a perturbed integrity of the thick filament (perturbed pattern of myomesin, MyBP-c, and MHC staining) suggesting that the thick filament perturbation may be a secondary effect of the disruption of titin’s M-line region [104]. This hypothesis is in accordance with the presence of myomesin close to MuRF1 binding domain, myomesin being the linker between titin and the thick filaments [104,180]. Accordingly, the thin filaments that interact with the stable N-terminal part of titin, were not affected by the over-expression of MuRF1 [104]. These results were further reinforced by the phenotype of transgenic mice expressing increased levels of MuRF1 in the heart (MuRF1-Tg+). Indeed, MuRF1-Tg+ mice presented a mild disruption of the M-line that was accentuated after TAC [122].

Moreover, very recent data showed that missense mutations in a titin Ig domain located in the M line–A-band transition zone of titin disrupted sarcomeres and led to HCM both in medaka fish and in humans [181]. In vitro studies revealed that these mutations increased the binding of MuRF1 [181]. This finding reinforces the idea that an increased titin-MuRF1 interaction favors cardiac sarcomere disruption (Figure 3 and Figure 4).

**Miscellaneous.** In mice over-expressing MuRF1 in skeletal muscle (TG-mice), two proteins were down-regulated, the α subunit of pyruvate dehydrogenase (PDH, α subunit E1) and its regulating kinase PDK2 [127]. PDH is a key enzyme complex that connects glycolysis with lipid oxidation, the citric acid cycle, and formation of ketone bodies. Inversely PDH and PDK2 were up-regulated in MuRF1-KO mice quadriceps, suggesting that PDH and PDK2 are negatively regulated in vivo by MuRF1. Although MuRF1 has been shown to interact with PDH in Y2H assay [127], no ubiquitination nor stability/destabilization assays were performed, and MuRF1 effect might be indirect. Indeed, PDH is a mitochondrial matrix enzyme and MuRF1 location in the mitochondria has to be ascertained. The MuRF1-PDH interaction may be an artifact that sometimes occurs in Y2H experiments [182]. Can MuRF1 and PDH proteins actually meet in the cell? This questioning is also legitimate for the other mitochondrial enzymes involved in energy metabolism that have been identified as potential MuRF1 partners using Y2H screen [21,31,127].

It should be noticed that an opposite action of MuRF1 on PDH was described in the heart. Indeed, MuRF1 over-expression in cardiomyocyte results in the inhibition of PPARa [174]. As PPARa is an indirect negative regulator of PDH, its inhibition by MuRF1 would result in an activation of PDH. This discrepancy with the study from Hirner and collaborators [127] might be explained by the difference of the approaches used (isolated cell vs. whole body context) or because differential metabolic and contractile properties (stimulation, speed of contraction, energy requirement, fatigability) between skeletal and cardiac muscles.

### 6.3. Ubiquitin Conjugating Enzymes, E2s

MuRF1 is a RING finger E3 ligase and thus exhibits no catalytic activity and relies entirely on its association with a specific E2 for the polyUb of specific substrates [183]. Like most E3 enzymes, MuRF1 is believed to interact with several E2 enzymes, which may form MuRF1-E2 couples specifically targeting dedicated substrates or family of substrates. However, the cognate E2s proteins of MuRF1 have long remained unknown or ambiguous. The first studies only relied on in vitro ubiquitination assays, using the UBE2D (formerly referred as UbCh5) family to ubiquitinate MuRF1 substrates like troponin I, MHC, a-actin, MCK, CnA, etc. [28,120,129,136,153]. Only recently, highly sensitive approaches (surface plasmon resonance (SPR) and split-GFP) reported that UBE2D2 does not physically interact with MuRF1 [38]. Moreover, an in cellulo degradation assay highlighted that UBE2D2 was not able to target alpha-actin and MHC in the presence of MuRF1, unlike five other E2s [138,184]. This confirmed that this UBE2D was not a physiological partner for MuRF1 in line with other reports on the UBE2D family. Indeed, UBE2D enzymes have intrinsic high catalytic activity in vitro without the presence of an E3 ligase and they also have the capacity to catalyze ubiquitin chains in vitro with almost any E3 ligase [183,185]. In vitro ubiquitination assays must therefore be cautiously used when addressing physiological E2/E3 couples.

Regarding physical interaction between MuRF1 and E2 proteins, several maps of human E2-RING E3 interactomes have been reported using Y2H approaches [186,187,188]. However, only one contained MuRF1 and experimental data did not fit with the conclusions made by the authors in the main text [186]. These interactomes revealed that most of the E3s almost exclusively interacted with proteins of the UBE2D or UBE2E families, while no E2 could be identified for many other E3s. This was later explained by studies showing that the majority of interactions between E3s and their cognate E2 exhibited low affinity and were labile (for a review see Stewart et al. 2016 [183]), which was the case for MuRF1 [138]. This implies that more sensitive and direct approaches than high throughput Y2H are needed to detect most E2-E3 interactions. For MuRF1, highly sensitive interactomic approaches using (SPR) and split-GFP were adapted to screen for muscle E2s interacting with MuRF1 [138]. This led to the identification of five UBE2s, namely E2E1, E2EG1, E2J1, E2J2, and E2L3, and revealed different affinities and kinetic parameters according to the UBE2-MuRF1 duo. These five UBE2-MuRF1 couples were functional since the presence of these E2s enhanced the degradation of telethonin by MuRF1 when co-expressed in HEK293T cells [138], UBE2D2 behaving as a negative control.

Interestingly, these studies, combined with known MuRF1 substrates and partners, raise the hypothesis that each UBE2-MuRF1 duo might be dedicated to a specific set of substrates. This means that depending on the E2 used, MuRF1 may have differential impacts on cellular metabolic pathways. Pioneering work has brought some line of evidence regarding these differential roles of the MuRF1-E2 couples. First, analysis of UBE2s-MuRF1 interactions in presence of telethonin revealed that this substrate stabilized and enhanced UBE2E1-MuRF1 and E2J1-MuRF1 interactions through an allosteric mechanism [138]. This means that a substrate can stabilize and thus favor the interaction between MuRF1 and a specific UBE2 enzyme. Second, this hypothesis was further supported by in cellulo degradation assay performed in HEK293T, showing that the UBE2E1-MuRF1 duo targets a-actin, but not MHCIIa, for subsequent degradation [184]. Further work is still needed to confirm this hypothesis, but this could represent a promising way to develop new drugs specifically protecting the contractile apparatus, while maintaining other MuRF1 functions.

## 7. MuRF1 Functions

### 7.1. Cardiac and Skeletal Muscle Functions

The generation of mice with a null deletion or over-expressing MuRF1/TRIM63 has allowed the investigation of the physiological functions of this E3 ligase. Numerous studies have supported a major role of MuRF1 in the development of skeletal muscle atrophy occurring during catabolic states (Figure 3). In the heart, analyses of MuRF1 mutants have highlighted a beneficial cardioprotective role of MuRF1 (Figure 4). First, MuRF1 inhibits injurious cardiac hypertrophy in response to pathological hypertrophic stimuli. Note that MuRF1 has also been reported to blunt physiological hypertrophy. Second, MuRF1 reduces cardiomyocytes apoptosis occurring after ischemia/reperfusion injury. Finally, MuRF1 could also be beneficial by promoting cardiac compensatory atrophy, to return to basal situation after cardiac hypertrophy provoked by pressure overload. Since MuRF1-KO mice have no phenotype in basal conditions neither in skeletal muscle nor in the heart, this suggests that MuRF1 alone is not sufficient to induce phenotypes described above, implying that MuRF1 is likely highly regulated and needs co-factors and regulatory proteins to function. In particular, E2 enzymes probably play a major role in this regulatory process.

The mechanisms through which MuRF1 induces skeletal muscle atrophy and prevents cardiac hypertrophy have not been fully deciphered yet. On one hand, MuRF1 displays identical mechanisms of action in both organs but with different goals. Indeed, MuRF1 is responsible for contractile proteins degradation in both organs and probably targets the same sarcomeric proteins. This leads in skeletal muscle to muscle atrophy and in the heart takes part in the anti-hypertrophic role of MuRF1. Gathering data from both organs allow to depict how MuRF1 may coordinate the breakdown of both thin and thick filaments (Figure 3; Figure 4). MuRF1 may act in two ways: 1) By binding to titin at the M-line, causing the disruption of titin/M-line interactions and hence leading to a partial disorganization of sarcomere assembly. 2) Once the sarcomere destabilized, MuRF1 will have access to contractile proteins (a-actin, troponins I, C and T, MHC, MLC, MyBP-C), for targeting them for ubiquitynation and proteasome-dependent degradation. On the other hand, MuRF1 may also have organ specific substrates to fulfill organ specific functions, such as c-Jun or CnA in the heart, whose degradation block cardiac hypertrophy (Figure 4). Similarly, the inhibition of protein synthesis has been reported both in skeletal and cardiac muscles. While investigations are still needed in skeletal muscle, in the heart MuRF1 has clearly been shown in several studies to limit the induction of hypertrophy-related genes in response to hypertrophic stimuli (Figure 4), involving three pathways (SRF, JNK, and AKT pathways). This inhibition of protein synthesis by MuRF1 could also explain how MuRF1 can limit cardiac hypertrophy.

### 7.2. Functional Redundancy of the MuRF Proteins

Functional redundancy between the three MuRF members has been reported. First, Y2H studies revealed that MuRF1 and MuRF2 can interact with several common proteins [21,31]. Both MuRF1 and MuRF3 target MHC for polyUb and degradation by the proteasome [129]. The MuRF1/MuRF3 double KO mice displayed skeletal muscle atrophy and cardiac hypertrophy, in standard condition, that is not detectable in the single MuRF-KO mice. These results suggested a functional redundancy between MuRF1 and MuRF3 for the degradation of the MHC and the development of this phenotype [129]. Moreover, non-targeted metabolomics analysis performed on the heart of the three MuRF-KO mice compared to WT mice revealed overlapping metabolic changes [189]. However, this study also revealed that each MuRF protein presented unique metabolic signatures suggesting that these proteins also have specific functions. This is further supported by the fact that each MuRF-KO mouse displayed different phenotype. For example, compared to WT and MuRF2-KO mice, MuRF1-KO mice spare skeletal muscle when submitted to a catabolic stress (for an example see [166]). MuRF1-KO mice also present an exaggerated cardiac hypertrophic response to pressure overload, which is not the case for the WT and MuRF2-KO mice [121]. Moreover, it was shown that several partners only interact with MuRF1 and not with its two relatives (e.g., Bif-1 or telethonin) [31,138,166]. Furthermore, some process alterations are found only in MuRF1-KO mice, for example the rescue of acetyl choline receptor (CHRN) half-life following a catabolic stress [166].

### 7.3. MuRF1 Dual Functions

Finally, even if MuRF1 has identical targets or regulates identical processes (contractile protein degradation or proteo-synthesis inhibition) in skeletal muscle and in the heart, the physiological impact is different. In the heart, cardiac hypertrophy is deleterious, and MuRF1 by limiting cardiac muscle growth appears to be a “beneficial” protein for the heart. While in skeletal muscle by controlling (in part) the same pathways, MuRF1 induces muscle atrophy during catabolic conditions, which is deleterious for skeletal muscle. It is noteworthy that at the whole organism level, MuRF1 appears to be a “beneficial” protein during transient catabolic state. Indeed, skeletal muscle represents about 40% of the body’s protein and is the major reservoir of amino acids mobilizable during pathologies or nutritional disorders for essential organs and functions (e.g., energetic purpose or the synthesis of acute phase proteins) [190,191]. However, during chronic catabolic states, the persistent degradation of contractile proteins by MuRF1 may lead to sustained muscle atrophy. In these conditions, MuRF1 becomes then a deleterious protein. This occurs in many diseases (cancer, sepsis, heart failure, kidney diseases, etc.) that are frequently associated with sustained muscle wasting (catabolic states). In this context, inhibiting MuRF1 appears as a good way to counteract skeletal muscle atrophy and several laboratories are trying to develop MuRF1 inhibitors.

## 8. MuRF1 Inhibitors/Modulators

Since the inhibition of MuRF1 (mutant studies) (cf § 5.1) has beneficial effects on the sparing of muscle mass during catabolic states, studies were conducted to identify pharmacological inhibitors of MuRF1. Ten years ago, a first small molecule was reported to inhibit MuRF1 auto-ubiquitination in vitro [185,192]. This MuRF1 inhibitor (P013222) displayed an EC_50_ around 2 µM and prevented the degradation of MHC in C2C12 myotubes subjected to Dex treatment (100 µM) [192]. However, no in vivo test was reported so far for this inhibitor. Moreover, it should be noted that the screening was based on in vitro ubiquitination assays performed with an E2 not physiologically interacting with MuRF1, which is questionable (see above, § 6.3).

More recently, another small-molecule (ID#704946) from an EMBL library including 130,000 compounds was reported to exhibit some MuRF1 inhibitory action [94]. The strategy was to inhibit the binding between the MuRF1 central domain and titin (Figure 1) to prevent the destabilization of titin by this MuRF1 domain. Among the 79 hits selected using the AlphaScreen^TM^ technology (Perkin Elmer), nine presented an EC_50_ < 25 µM including one that was also able to block in vitro MuRF1 auto-ubiquitination. Surprisingly, this molecule was also able to inhibit the mRNA expression of MuRF1. The efficiency of ID#704946 was tested in vivo, in mice, using monocrotaline (MCT) injections over six weeks, resulting in both cardiac and skeletal muscle cachexia. However, ID#704946 was inefficient for heart protection as mice fed an ID#704946-supplemented diet displayed similar cardiac patho-physiological alterations when compared to MCT mice fed a normal chow. Regarding skeletal muscles, no protective effect was observed on EDL and soleus muscles while a mild protective effect was reported on tibialis anterior mass and on the contractile function of diaphragm [94]. The mechanisms by which ID#704946 decreases mRNA levels is still not understood but this may account for the observed muscle improvements.

The efficiency of ID#704946 compound was further analyzed following a myocardial infarction-induced heart failure model leading to diaphragm myopathy [193]. The authors confirmed that ID#704946 compound ameliorates contractile properties of the diaphragm, i.e., the maximal force and the peak power but without improvement on fiber atrophy. They also confirmed that this compound inhibited MuRF1 expression but also MuRF2 expression [193]. Knowing that MuRF1-MuRF2 double-KO has adverse effects on mice, future development of MuRF1 inhibitory molecules should focus on more specific compounds for avoiding long-term side effects.

These pioneer studies opened the way for MuRF1 inhibition to treat muscle atrophy, but they also highlighted the long and difficult path before obtaining an efficient compound with no side effect. We know that MuRF1 has several targets belonging to different metabolic pathways in different muscles (cardiac, skeletal, smooth), which brings us to a crucial question: Is it wise to inhibit all the functions of MuRF1 in any type of muscle? Only deep investigations will provide an answer but deciphering the role of the MuRF1 partners will probably be mandatory as their interaction with MuRF1 may directly impact the degradation of specific substrates or set of substrates.

Moreover, it must be kept in mind that MuRF1 has a beneficial effect in the heart and therefore MuRF1 should only be inhibited in skeletal muscle. This implies to selectively target the drug to skeletal muscle. Another point to consider when developing inhibitors, is that MuRF1 belongs to a family including two close relatives, MuRF2 and MuRF3, sharing redundant and non-redundant functions. Thus, inhibitors should be highly specific to only inhibit skeletal muscle sarcomeric protein degradation while maintaining other MuRFs functions.

## 9. Conclusions

Maintaining the structure and function of striated muscles during pathologies is a tremendous public health issue. Despite this importance, how the sarcomere assembly and disassembly occur is still a poorly resolved enigma. We have known for decades that the UPS plays an important role in the degradation of long-lived proteins such as components of the contractile apparatus of striated muscles, but the exact sequence of events is unknown.

In this context, the E3 ubiquitin ligase of the TRIM family MuRF1/TRIM63 has aroused growing interest for its key role in the development of skeletal muscle atrophy. As such, MuRF1 appeared as an appealing target for the development of drugs dedicated to the preservation of muscle mass. However, while initially described as a restricted contractile protein manipulator, more recent data suggest that this E3 ligase plays on different fronts, e.g., by targeting pro-anabolic factors like CnA. The expanding panel of targets may complicate the therapeutic strategies as one can wonder whether inhibiting the degradation of all the MuRF1 targets is suitable. On the one hand it may help having a coordinated action but on the other hand it may affect other processes (e.g., the intracellular trafficking) that could lower the efficiency of the inhibitor or affect cellular homeostasis. Besides the targets of MuRF1 that undergo proteasome degradation, several proteins are “just” partners of MuRF1, the question being: For what purpose? Except for titin, almost nothing is known about the underlying mechanisms linked to the interaction between MuRF1 and these partners. This is notably the case for the heterodimerization of MuRF1 with MuRF2 and MuRF3. Whether these MuRF1 isoforms (and other partners) are needed for some MuRF1 activities and/or exhibit redundant functions is probably an important aspect for strategies aiming at the development of new drugs.

Another important aspect is the physiological importance of MuRF1 in the different muscle types (cardiac, skeletal, and smooth). The available data are in favor of a similar impact of MuRF1 on contractile proteins, i.e., enhanced degradation. However, physiologically, MuRF1 is protective in the heart by limiting deleterious cardiac hypertrophy while it is the opposite in skeletal muscle where muscle loss weakens the organism. This could be tricky for the development of MuRF1 inhibitors as improving skeletal muscle mass might be deleterious for cardiac function. More work is clearly needed for clarifying the role of MuRF1 in these different muscles, keeping in mind that almost nothing is known about smooth muscles. One possibility to avoid such problems would be to play on more specific roles of MuRF1 by targeting the interaction between MuRF1 and some partners. Such a strategy may help in finding more specific pharmacological drugs and also should avoid unwanted inhibition of MuRF2 or MuRF3. Among the different possibilities, including the E2 enzymes in the game may prove to be highly interesting, either MuRF1-E2 or more largely MuRF1-E2-substrate; this may limit the number of targets affected by the future drugs. Identifying the limiting steps that cause skeletal muscle atrophy may also help with designing future drugs. On the whole, many pitfalls remain to be circumvented before finding molecules both efficient and safe for human application. Future work should focus on (i) the involvement of MuRF1 in the series of event leading to contractile protein loss, (ii) distinguishing the different roles of MuRF1 in skeletal and cardiac muscle, and (iii) the interplay between MuRF1 and its partners (including MuRFs isoforms). Such studies may prove to be highly valuable for reaching the final goal, i.e., designing new drugs to maintain muscle mass in patients suffering from various pathologies.

## Figures and Tables

**Table 1:** Location of different MuRF1 truncated forms.

MuRF1 Region	Location	References
Central region (87–231)	M line	[2,104]
COS domain	Z line and I band	[27]
MuRF1-delta COS	Cytosol	[105]
RING domain	Nucleus	[104]
MuRF1-delta RING	Cytosol and M line	[106]

## Abbreviations

**a-actin**	alpha-actin
**ActRIIB**	activin receptor type 2B
**ALS**	amyotrophic lateral sclerosis
**Ang II**	angiotensin II
**ANP**	atrial natriuretic peptide
**AT**	acidic tail
**AT1R**	angiotensin II receptor type 1
**Bb2**	B-box type 2 domain
**bHLH**	basic helix-loop-helix
**Bif-1**	BAX interacting factor 1
**C/EBP**	CCAAT/enhancer binding protein
**CARP**	ubiquitin carboxyl-terminal hydrolase MINDY-3
**CC**	coiled-coil domain
**CHOP**	C/EBP homologous protein
**CHRN**	cholinergic receptor nicotinic/nicotinic acetylcholine receptor
**CK**	creatine kinase
**cMyBP-C**	cardiac MyBP-C isoform
**can**	calcineurin
**COS box**	C-terminal subgroup one signature-box
**CRL4**	Cullin-RING-based E3 ubiquitin-protein ligase 4
**CSA**	cross-sectional area; **Dex**, dexamethasone
**dKO**	double knock-out
**EDL**	extensor digitorium longus muscle
**EndoB1**	endophilin B1
**EPR**	electron paramagnetic resonance
**FoxO**	forkhead box family of transcription factors
**GME**	glucocorticoid modulatory elements
**GMEB1**	glucocorticoid modulatory element binding protein-1
**GR**	glucocorticoid receptor
**HCM**	hypertrophic cardiomyopathy
**HD**	helical domain
**HIBADH**	3-hydroxyisobutyrate dehydrogenase
**ICU**	intensive care unit
**IGF-1**	insulin-like growth factor 1
**IL1**	interleukine 1
**I/R**	ischemia/reperfusion
**JNK**	c-Jun N-terminal kinases
**KO**	knock-out
**LAMP1**	lyososomal-associated membrane protein 1
**LC3**	microtubule-associated proteins 1A/1B light chain 3b
**M1/M2 dKO**	homozygous constitutive null mutants for MuRF1 or MuRF2
**MAFbx**	muscle atrophy F-box protein
**MCT**	monocrotaline
**MEF2**	myocyte enhancer factor-2
**MFC**	MuRF family specific motif
**MHC**	myosin heavy chain
**MHCIIa**	myosin heavy chain type IIa
**MI**	myocardial infarction
**MLC**	myosin light chain
**MLP/Csrp3**	muscle limp protein/cystine and glycine-rich protein 3
**MCK**	muscle-type creatine kinase
**mTORC1**	mammalian target of rapamycin complex 1
**MuRF**	muscle-specific RING finger protein
**MuRF1-KO**	transgenic mice specifically silenced for MuRF1
**MuRF1-TG**	transgenic mice specifically over-expressing MuRF1 in skeletal muscle
**MuRF1-Tg+**	transgenic mice expressing increased levels of MuRF1 specifically in the heart
**MuRF1-** **ΔRING**	transgenic mice with a mutant dominant negative form of MuRF1 corresponding to a RING-deletion of MuRF1
**MyBP-C**	myosin binding prot-C
**MHC**	myosin heavy chain
**NF-kB**	nuclear factor kappa-light-chain-enhancer of activated B cells
**NFAT**	nuclear factor of activated T cells
**NRVM**	neonatal rat ventricular myocytes
**O-MCK**	oxidized form of MCK
**PE**	phenylephrine
**PDH**	pyruvate dehydrogenase
**PDK2**	pyruvate dehydrogenase kinase isoform 2
**PI3K**	phosphoinositide 3-kinase
**polyUb**	polyubiquitination
**PMA**	plasma membrane ATPase
**PPARα**	peroxisome proliferator activated receptors
**PPRE**	PPAR response element
**RACK1**	receptor of activated protein C kinase 1
**RBCC**	RING-B box-coiled-coil
**RING**	really interesting new gene
**ROS**	reactive oxygen species
**SPR**	surface plasmon resonance
**SQTM1/p62**	Sequestosome-1
**SRF**	serum response factor
**SUMO**	small ubiquitin-related modifier
**TAC**	transaortic constriction model
**TFEB**	transcription factor EB
**TNF**	transforming growth factor
**TnnI, TnnC**	troponin I and C
**TRAF6**	TNF receptor associated factors 6
**TRIP12**	thyroid receptor-interacting protein 12
**TRIM**	tripartite motif family
**UPS**	ubiquitin-proteasome system
**WT**	wild-type
**Y2H**	yeast two-hybrid.

## References

[1] Zheng N., Shabek N. (2017). Ubiquitin Ligases: Structure, Function, and Regulation. Annu. Rev. Biochem..

[2] Centner T., Yano J., Kimura E., McElhinny A.S., Pelin K., Witt C.C., Bang M.L., Trombitas K., Granzier H., Gregorio C.C. (2001). Identification of muscle specific ring finger proteins as potential regulators of the titin kinase domain. J. Mol. Biol..

[3] Bodine S.C., Latres E., Baumhueter S., Lai V.K., Nunez L., Clarke B.A., Poueymirou W.T., Panaro F.J., Na E., Dharmarajan K. (2001). Identification of ubiquitin ligases required for skeletal muscle atrophy. Science.

[4] Taillandier D., Polge C. (2019). Skeletal muscle atrogenes: From rodent models to human pathologies. Biochimie.

[5] Reddy B.A., Etkin L.D., Freemont P.S. (1992). A novel zinc finger coiled-coil domain in a family of nuclear proteins. Trends Biochem. Sci..

[6] Meroni G., Diez-Roux G. (2005). TRIM/RBCC, a novel class of ‘single protein RING finger’ E3 ubiquitin ligases. BioEssays.

[7] Reymond A., Meroni G., Fantozzi A., Merla G., Cairo S., Luzi L., Riganelli D., Zanaria E., Messali S., Cainarca S. (2001). The tripartite motif family identifies cell compartments. EMBO J..

[8] Short K.M., Cox T.C. (2006). Subclassification of the RBCC/TRIM superfamily reveals a novel motif necessary for microtubule binding. J. Biol. Chem..

[9] Caratozzolo M.F., Marzano F., Mastropasqua F., Sbisà E., Tullo A. (2017). TRIM8: Making the Right Decision between the Oncogene and Tumour Suppressor Role. Genes.

[10] Borlepawar A., Frey N., Rangrez A.Y. (2019). A systematic view on E3 ligase Ring TRIMmers with a focus on cardiac function and disease. Trends Cardiovasc. Med..

[11] Sardiello M., Cairo S., Fontanella B., Ballabio A., Meroni G. (2008). Genomic analysis of the TRIM family reveals two groups of genes with distinct evolutionary properties. BMC Evol. Biol..

[12] Carthagena L., Bergamaschi A., Luna J.M., David A., Uchil P.D., Margottin-Goguet F., Mothes W., Hazan U., Transy C., Pancino G. (2009). Human TRIM Gene Expression in Response to Interferons. PLoS ONE.

[13] Marín I. (2012). Origin and Diversification of TRIM Ubiquitin Ligases. PLoS ONE.

[14] Ozato K., Shin D.-M., Chang T.-H., Morse H.C. (2008). TRIM family proteins and their emerging roles in innate immunity. Nat. Rev. Immunol..

[15] Deshaies R.J., Joazeiro C.A.P. (2009). RING Domain E3 Ubiquitin Ligases. Annu. Rev. Biochem..

[16] Esposito D., Koliopoulos M.G., Rittinger K. (2017). Structural determinants of TRIM protein function. Biochem. Soc. Trans..

[17] Mrosek M., Meier S., Ucurum-Fotiadis Z., von Castelmur E., Hedbom E., Lustig A., Grzesiek S., Labeit D., Labeit S., Mayans O. (2008). Structural analysis of B-Box 2 from MuRF1: Identification of a novel self-association pattern in a RING-like fold. Biochemistry.

[18] Cassandri M., Smirnov A., Novelli F., Pitolli C., Agostini M., Malewicz M., Melino G., Raschellà G. (2017). Zinc-finger proteins in health and disease. Cell Death Discov..

[19] Budhidarmo R., Nakatani Y., Day C.L. (2012). RINGs hold the key to ubiquitin transfer. Trends Biochem. Sci..

[20] Li Y., Wu H., Wu W., Zhuo W., Liu W., Zhang Y., Cheng M., Chen Y.-G., Gao N., Yu H. (2014). Structural insights into the TRIM family of ubiquitin E3 ligases. Cell Res..

[21] Witt S.H., Granzier H., Witt C.C., Labeit S. (2005). MURF-1 and MURF-2 target a specific subset of myofibrillar proteins redundantly: Towards understanding MURF-dependent muscle ubiquitination. J. Mol. Biol..

[22] Li H.-H., Du J., Fan Y.-N., Zhang M.-L., Liu D.-P., Li L., Lockyer P., Kang E.Y., Patterson C., Willis M.S. (2011). The ubiquitin ligase MuRF1 protects against cardiac ischemia/reperfusion injury by its proteasome-dependent degradation of phospho-c-Jun. Am. J. Pathol..

[23] Stevens M., Franke B., Skorupka K.A., Cafiso D.S., Pornillos O., Mayans O., Norman D.G. (2019). Exploration of the TRIM Fold of MuRF1 Using EPR Reveals a Canonical Antiparallel Structure and Extended COS-Box. J. Mol. Biol..

[24] Spencer J.A., Eliazer S., Ilaria R.L., Richardson J.A., Olson E.N. (2000). Regulation of microtubule dynamics and myogenic differentiation by MURF, a striated muscle RING-finger protein. J. Cell Biol..

[25] Wagner J.M., Roganowicz M.D., Skorupka K., Alam S.L., Christensen D., Doss G., Wan Y., Frank G.A., Ganser-Pornillos B.K., Sundquist W.I. (2016). Mechanism of B-box 2 domain-mediated higher-order assembly of the retroviral restriction factor TRIM5α. Elife.

[26] Cao T., Borden K.L., Freemont P.S., Etkin L.D. (1997). Involvement of the rfp tripartite motif in protein-protein interactions and subcellular distribution. J. Cell Sci..

[27] Franke B., Gasch A., Rodriguez D., Chami M., Khan M.M., Rudolf R., Bibby J., Hanashima A., Bogomolovas J., von Castelmur E. (2014). Molecular basis for the fold organization and sarcomeric targeting of the muscle atrogin MuRF1. Open Biol..

[28] Kedar V., McDonough H., Arya R., Li H.-H., Rockman H.A., Patterson C. (2004). Muscle-specific RING finger 1 is a bona fide ubiquitin ligase that degrades cardiac troponin I. Proc. Natl. Acad. Sci. USA.

[29] Koyama S., Hata S., Witt C.C., Ono Y., Lerche S., Ojima K., Chiba T., Doi N., Kitamura F., Tanaka K. (2008). Muscle RING-finger protein-1 (MuRF1) as a connector of muscle energy metabolism and protein synthesis. J. Mol. Biol..

[30] Gundogdu M., Walden H. (2019). Structural basis of generic versus specific E2–RING E3 interactions in protein ubiquitination. Protein Sci..

[31] Witt C.C., Witt S.H., Lerche S., Labeit D., Back W., Labeit S. (2008). Cooperative control of striated muscle mass and metabolism by MuRF1 and MuRF2. EMBO J..

[32] Perera S., Mankoo B., Gautel M. (2012). Developmental regulation of MURF E3 ubiquitin ligases in skeletal muscle. J. Muscle Res. Cell Motil..

[33] Pizon V., Iakovenko A., van der Ven P.F.M., Kelly R., Fatu C., Fürst D.O., Karsenti E., Gautel M. (2002). Transient association of titin and myosin with microtubules in nascent myofibrils directed by the MURF2 RING-finger protein. J. Cell Sci..

[34] Ochala J., Gustafson A.-M., Diez M.L., Renaud G., Li M., Aare S., Qaisar R., Banduseela V.C., Hedström Y., Tang X. (2011). Preferential skeletal muscle myosin loss in response to mechanical silencing in a novel rat intensive care unit model: Underlying mechanisms. J. Physiol..

[35] Nguyen T., Bowen T.S., Augstein A., Schauer A., Gasch A., Linke A., Labeit S., Adams V. (2020). Expression of MuRF1 or MuRF2 is essential for the induction of skeletal muscle atrophy and dysfunction in a murine pulmonary hypertension model. Skelet. Muscle.

[36] Lecker S.H., Jagoe R.T., Gilbert A., Gomes M., Baracos V., Bailey J., Price S.R., Mitch W.E., Goldberg A.L. (2004). Multiple types of skeletal muscle atrophy involve a common program of changes in gene expression. FASEB J..

[37] Jagoe R.T., Lecker S.H., Gomes M., Goldberg A.L. (2002). Patterns of gene expression in atrophying skeletal muscles: Response to food deprivation. FASEB J..

[38] Polge C., Koulmann N., Claustre A., Jarzaguet M., Serrurier B., Combaret L., Béchet D., Bigard X., Attaix D., Taillandier D. (2016). UBE2D2 is not involved in MuRF1-dependent muscle wasting during hindlimb suspension. Int. J. Biochem. Cell Biol..

[39] Seaborne R.A., Hughes D.C., Turner D.C., Owens D.J., Baehr L.M., Gorski P., Semenova E.A., Borisov O.V., Larin A.K., Popov D.V. (2019). UBR5 is a novel E3 ubiquitin ligase involved in skeletal muscle hypertrophy and recovery from atrophy. J. Physiol..

[40] Pinheiro-Dardis C.M., Erbereli B.T., Gigo-Benato D., Castro P.A.T.S., Russo T.L. (2017). Electrical stimulation delays reinnervation in denervated rat muscle. Muscle Nerve.

[41] Fisher A.G., Seaborne R.A., Hughes T.M., Gutteridge A., Stewart C., Coulson J.M., Sharples A.P., Jarvis J.C. (2017). Transcriptomic and epigenetic regulation of disuse atrophy and the return to activity in skeletal muscle. FASEB J..

[42] Baptista I.L., Leal M.L., Artioli G.G., Aoki M.S., Fiamoncini J., Turri A.O., Curi R., Miyabara E.H., Moriscot A.S. (2010). Leucine attenuates skeletal muscle wasting via inhibition of ubiquitin ligases. Muscle Nerve.

[43] Baptista I.L., Silva W.J., Artioli G.G., Guilherme J.P.L.F., Leal M.L., Aoki M.S., Miyabara E.H., Moriscot A.S. (2013). Leucine and HMB differentially modulate proteasome system in skeletal muscle under different sarcopenic conditions. PLoS ONE.

[44] Baptista I.L., Silvestre J.G., Silva W.J., Labeit S., Moriscot A.S. (2017). FoxO3a suppression and VPS34 activity are essential to anti-atrophic effects of leucine in skeletal muscle. Cell Tissue Res..

[45] Sacheck J.M., Ohtsuka A., McLary S.C., Goldberg A.L. (2004). IGF-I stimulates muscle growth by suppressing protein breakdown and expression of atrophy-related ubiquitin ligases, atrogin-1 and MuRF1. Am. J. Physiol. Endocrinol. Metab..

[46] Schakman O., Kalista S., Bertrand L., Lause P., Verniers J., Ketelslegers J.M., Thissen J.P. (2008). Role of Akt/GSK-3beta/beta-catenin transduction pathway in the muscle anti-atrophy action of insulin-like growth factor-I in glucocorticoid-treated rats. Endocrinology.

[47] Cleveland B.M., Weber G.M., Blemings K.P., Silverstein J.T. (2009). Insulin-like growth factor-I and genetic effects on indexes of protein degradation in response to feed deprivation in rainbow trout (Oncorhynchus mykiss). Am. J. Physiol. Regul. Integr. Comp. Physiol..

[48] Song J., Baer L.A., Threlkeld M.R.S., Geng C., Wade C.E., Wolf S.E. (2019). Insulin and exercise improved muscle function in rats with severe burns and hindlimb unloading. Physiol. Rep..

[49] Yoshida T., Semprun-Prieto L., Sukhanov S., Delafontaine P. (2010). IGF-1 prevents ANG II-induced skeletal muscle atrophy via Akt- and Foxo-dependent inhibition of the ubiquitin ligase atrogin-1 expression. Am. J. Physiol. Heart Circ. Physiol..

[50] Schakman O., Dehoux M., Bouchuari S., Delaere S., Lause P., Decroly N., Shoelson S.E., Thissen J.-P. (2012). Role of IGF-I and the TNFα/NF-κB pathway in the induction of muscle atrogenes by acute inflammation. Am. J. Physiol. Endocrinol. Metab..

[51] Cohen S., Nathan J.A., Goldberg A.L. (2015). Muscle wasting in disease: Molecular mechanisms and promising therapies. Nat. Rev. Drug Discov..

[52] Furuyama T., Kitayama K., Yamashita H., Mori N. (2003). Forkhead transcription factor FOXO1 (FKHR)-dependent induction of PDK4 gene expression in skeletal muscle during energy deprivation. Biochem. J..

[53] Kwon H.-S., Huang B., Unterman T.G., Harris R.A. (2004). Protein Kinase B-α Inhibits Human Pyruvate Dehydrogenase Kinase-4 Gene Induction by Dexamethasone Through Inactivation of FOXO Transcription Factors. Diabetes.

[54] Waddell D.S., Baehr L.M., van den Brandt J., Johnsen S.A., Reichardt H.M., Furlow J.D., Bodine S.C. (2008). The glucocorticoid receptor and FOXO1 synergistically activate the skeletal muscle atrophy-associated MuRF1 gene. Am. J. Physiol. Endocrinol. Metab..

[55] Smith I.J., Alamdari N., O’Neal P., Gonnella P., Aversa Z., Hasselgren P.-O. (2010). Sepsis increases the expression and activity of the transcription factor Forkhead Box O 1 (FOXO1) in skeletal muscle by a glucocorticoid-dependent mechanism. Int. J. Biochem. Cell Biol..

[56] McLoughlin T.J., Smith S.M., DeLong A.D., Wang H., Unterman T.G., Esser K.A. (2009). FoxO1 induces apoptosis in skeletal myotubes in a DNA-binding-dependent manner. Am. J. Physiol. Cell Physiol..

[57] Xu J., Li R., Workeneh B., Dong Y., Wang X., Hu Z. (2012). Transcription factor FoxO1, the dominant mediator of muscle wasting in chronic kidney disease, is inhibited by microRNA-486. Kidney Int..

[58] Goodman C.A., McNally R.M., Hoffmann F.M., Hornberger T.A. (2013). Smad3 induces atrogin-1, inhibits mTOR and protein synthesis, and promotes muscle atrophy in vivo. Mol. Endocrinol..

[59] Bollinger L.M., Witczak C.A., Houmard J.A., Brault J.J. (2014). SMAD3 augments FoxO3-induced MuRF-1 promoter activity in a DNA-binding-dependent manner. Am. J. Physiol. Cell Physiol..

[60] Kang S.-H., Lee H.-A., Kim M., Lee E., Sohn U.D., Kim I. (2017). Forkhead box O_3_ plays a role in skeletal muscle atrophy through expression of E3 ubiquitin ligases MuRF-1 and atrogin-1 in Cushing’s syndrome. Am. J. Physiol. Endocrinol. Metab..

[61] Milan G., Romanello V., Pescatore F., Armani A., Paik J.-H., Frasson L., Seydel A., Zhao J., Abraham R., Goldberg A.L. (2015). Regulation of autophagy and the ubiquitin–proteasome system by the FoxO transcriptional network during muscle atrophy. Nat. Commun..

[62] Moylan J.S., Smith J.D., Chambers M.A., McLoughlin T.J., Reid M.B. (2008). TNF induction of atrogin-1/MAFbx mRNA depends on Foxo4 expression but not AKT-Foxo1/3 signaling. Am. J. Physiol. Cell Physiol..

[63] O’Neill B.T., Bhardwaj G., Penniman C.M., Krumpoch M.T., Beltran P.A.S., Klaus K., Poro K., Li M., Pan H., Dreyfuss J.M. (2019). FoxO Transcription Factors Are Critical Regulators of Diabetes-Related Muscle Atrophy. Diabetes.

[64] Puigserver P., Rhee J., Donovan J., Walkey C.J., Yoon J.C., Oriente F., Kitamura Y., Altomonte J., Dong H., Accili D. (2003). Insulin-regulated hepatic gluconeogenesis through FOXO1–PGC-1α interaction. Nature.

[65] Sandri M., Lin J., Handschin C., Yang W., Arany Z.P., Lecker S.H., Goldberg A.L., Spiegelman B.M. (2006). PGC-1α protects skeletal muscle from atrophy by suppressing FoxO_3_ action and atrophy-specific gene transcription. Proc. Natl. Acad. Sci. USA.

[66] Watson M.L., Baehr L.M., Reichardt H.M., Tuckermann J.P., Bodine S.C., Furlow J.D. (2012). A cell-autonomous role for the glucocorticoid receptor in skeletal muscle atrophy induced by systemic glucocorticoid exposure. Am. J. Physiol. Endocrinol. Metab..

[67] Wu C.-L., Cornwell E.W., Jackman R.W., Kandarian S.C. (2014). NF-κB but not FoxO sites in the MuRF1 promoter are required for transcriptional activation in disuse muscle atrophy. Am. J. Physiol. Cell Physiol..

[68] Yarar-Fisher C., Bickel C.S., Kelly N.A., Stec M.J., Windham S.T., McLain A.B., Oster R.A., Bamman M.M. (2016). Heightened TWEAK-NF-κB signaling and inflammation-associated fibrosis in paralyzed muscles of men with chronic spinal cord injury. Am. J. Physiol. Endocrinol. Metab..

[69] Vainshtein A., Sandri M. (2020). Signaling Pathways that Control Muscle Mass. Int. J. Mol. Sci..

[70] Files D.C., D’Alessio F.R., Johnston L.F., Kesari P., Aggarwal N.R., Garibaldi B.T., Mock J.R., Simmers J.L., DeGorordo A., Murdoch J. (2012). A critical role for muscle ring finger-1 in acute lung injury-associated skeletal muscle wasting. Am. J. Respir. Crit. Care Med..

[71] Sriram S., Subramanian S., Juvvuna P.K., Ge X., Lokireddy S., McFarlane C.D., Wahli W., Kambadur R., Sharma M. (2014). Myostatin augments muscle-specific ring finger protein-1 expression through an NF-kB independent mechanism in SMAD3 null muscle. Mol. Endocrinol..

[72] Arrowsmith C.H. (1999). Structure and function in the p53 family. Cell Death Differ..

[73] Benosman S., Meng X., Grabowiecki Y.V., Palamiuc L., Hritcu L., Gross I., Mellitzer G., Taya Y., Loeffler J.-P., Gaiddon C. (2011). Complex regulation of p73 isoforms after alteration of the amyloid precursor polypeptide (APP) function and DNA damages in neurons. J. Biol. Chem..

[74] Menendez D., Inga A., Resnick M.A. (2009). The expanding universe of p53 targets. Nat. Rev. Cancer.

[75] Von Grabowiecki Y., Abreu P., Blanchard O., Palamiuc L., Benosman S., Mériaux S., Devignot V., Gross I., Mellitzer G., de Aguilar J.L.G. (2016). Transcriptional activator TAp63 is upregulated in muscular atrophy during ALS and induces the pro-atrophic ubiquitin ligase Trim63. Elife.

[76] Sardiello M., Palmieri M., di Ronza A., Medina D.L., Valenza M., Gennarino V.A., di Malta C., Donaudy F., Embrione V., Polishchuk R.S. (2009). A gene network regulating lysosomal biogenesis and function. Science.

[77] Palmieri M., Impey S., Kang H., di Ronza A., Pelz C., Sardiello M., Ballabio A. (2011). Characterization of the CLEAR network reveals an integrated control of cellular clearance pathways. Hum. Mol. Genet..

[78] Bois P.D., Tortola C.P., Lodka D., Kny M., Schmidt F., Song K., Schmidt S., Bassel-Duby R., Olson E.N., Fielitz J. (2015). Angiotensin II Induces Skeletal Muscle Atrophy by Activating TFEB-Mediated MuRF1 Expression. Circ. Res..

[79] Hasty P., Bradley A., Morris J.H., Edmondson D.G., Venuti J.M., Olson E.N., Klein W.H. (1993). Muscle deficiency and neonatal death in mice with a targeted mutation in the myogenin gene. Nature.

[80] Nabeshima Y., Hanaoka K., Hayasaka M., Esuml E., Li S., Nonaka I., Nabeshima Y. (1993). Myogenin gene disruption results in perinatal lethality because of severe muscle defect. Nature.

[81] Moresi V., Williams A.H., Meadows E., Flynn J.M., Potthoff M.J., McAnally J., Shelton J.M., Backs J., Klein W.H., Richardson J.A. (2010). Myogenin and class II HDACs control neurogenic muscle atrophy by inducing E3 ubiquitin ligases. Cell.

[82] Ninfali C., Siles L., Darling D.S., Postigo A. (2018). Regulation of muscle atrophy-related genes by the opposing transcriptional activities of ZEB1/CtBP and FOXO3. Nucleic Acids Res..

[83] Nerlov C. (2007). The C/EBP family of transcription factors: A paradigm for interaction between gene expression and proliferation control. Trends Cell Biol..

[84] Gonnella P., Alamdari N., Tizio S., Aversa Z., Petkova V., Hasselgren P.-O. (2011). C/EBPβ regulates dexamethasone-induced muscle cell atrophy and expression of atrogin-1 and MuRF1. J. Cell. Biochem..

[85] Ding H., Zhang G., Sin K.W.T., Liu Z., Lin R.-K., Li M., Li Y.-P. (2017). Activin A induces skeletal muscle catabolism via p38β mitogen-activated protein kinase. J. Cachexia Sarcopenia Muscle.

[86] Silva K.A.S., Dong J., Dong Y., Dong Y., Schor N., Tweardy D.J., Zhang L., Mitch W.E. (2015). Inhibition of Stat3 Activation Suppresses Caspase-3 and the Ubiquitin-Proteasome System, Leading to Preservation of Muscle Mass in Cancer Cachexia. J. Biol. Chem..

[87] Fang C.X., Dong F., Thomas D.P., Ma H., He L., Ren J. (2008). Hypertrophic cardiomyopathy in high-fat diet-induced obesity: Role of suppression of forkhead transcription factor and atrophy gene transcription. Am. J. Physiol. Heart Circ. Physiol..

[88] Shimizu H., Langenbacher A.D., Huang J., Wang K., Otto G., Geisler R., Wang Y., Chen J.-N. (2017). The Calcineurin-FoxO-MuRF1 signaling pathway regulates myofibril integrity in cardiomyocytes. Elife.

[89] Baskin K.K., Taegtmeyer H. (2011). AMP-activated protein kinase regulates E3 ligases in rodent heart. Circ. Res..

[90] Jaitovich A., Angulo M., Lecuona E., Dada L.A., Welch L.C., Cheng Y., Gusarova G., Ceco E., Liu C., Shigemura M. (2015). High CO_2_ levels cause skeletal muscle atrophy via AMP-activated kinase (AMPK), FoxO3a protein, and muscle-specific Ring finger protein 1 (MuRF1). J. Biol. Chem..

[91] Namuduri A.V., Heras G., Mi J., Cacciani N., Hörnaeus K., Konzer A., Lind S.B., Larsson L., Gastaldello S. (2017). A Proteomic Approach to Identify Alterations in the Small Ubiquitin-like Modifier (SUMO) Network during Controlled Mechanical Ventilation in Rat Diaphragm Muscle. Mol. Cell. Proteom..

[92] Heras G., Namuduri A.V., Traini L., Shevchenko G., Falk A., Lind S.B., Jia M., Tian G., Gastaldello S. (2019). Muscle RING-finger protein-1 (MuRF1) functions and cellular localization are regulated by SUMO1 post-translational modification. J. Mol. Cell Biol..

[93] Kim H.T., Kim K.P., Lledias F., Kisselev A.F., Scaglione K.M., Skowyra D., Gygi S.P., Goldberg A.L. (2007). Certain pairs of ubiquitin-conjugating enzymes (E2s) and ubiquitin-protein ligases (E3s) synthesize nondegradable forked ubiquitin chains containing all possible isopeptide linkages. J. Biol. Chem..

[94] Bowen T.S., Adams V., Werner S., Fischer T., Vinke P., Brogger M.N., Mangner N., Linke A., Sehr P., Lewis J. (2017). Small-molecule inhibition of MuRF1 attenuates skeletal muscle atrophy and dysfunction in cardiac cachexia. J. Cachexia Sarcopenia Muscle.

[95] Mota R., Rodríguez J.E., Bonetto A., O’Connell T.M., Asher S.A., Parry T.L., Lockyer P., McCudden C.R., Couch M.E., Willis M.S. (2017). Post-translationally modified muscle-specific ubiquitin ligases as circulating biomarkers in experimental cancer cachexia. Am. J. Cancer Res..

[96] Bdolah Y., Segal A., Tanksale P., Karumanchi S.A., Lecker S.H. (2007). Atrophy-related ubiquitin ligases atrogin-1 and MuRF-1 are associated with uterine smooth muscle involution in the postpartum period. Am. J. Physiol. Regul. Integr. Comp. Physiol..

[97] Schiaffino S., Dyar K.A., Ciciliot S., Blaauw B., Sandri M. (2013). Mechanisms regulating skeletal muscle growth and atrophy. FEBS J..

[98] Schiaffino S., Reggiani C. (2011). Fiber types in mammalian skeletal muscles. Physiol. Rev..

[99] Moriscot A.S., Baptista I.L., Bogomolovas J., Witt C., Hirner S., Granzier H., Labeit S. (2010). MuRF1 is a muscle fiber-type II associated factor and together with MuRF2 regulates type-II fiber trophicity and maintenance. J. Struct. Biol..

[100] Polge C., Leulmi R., Jarzaguet M., Claustre A., Combaret L., Béchet D., Heng A.-E., Attaix D., Taillandier D. (2016). UBE2B is implicated in myofibrillar protein loss in catabolic C2C12 myotubes: UBE2B and myofibrillar protein degradation. J. Cachexia Sarcopenia Muscle.

[101] Belova S.P., Mochalova E.P., Kostrominova T.Y., Shenkman B.S., Nemirovskaya T.L. (2020). P38α-MAPK Signaling Inhibition Attenuates Soleus Atrophy during Early Stages of Muscle Unloading. Int. J. Mol. Sci..

[102] Macpherson P.C.D., Wang X., Goldman D. (2011). Myogenin regulates denervation-dependent muscle atrophy in mouse soleus muscle. J. Cell. Biochem..

[103] Atherton P.J., Greenhaff P.L., Phillips S.M., Bodine S.C., Adams C.M., Lang C.H. (2016). Control of skeletal muscle atrophy in response to disuse: Clinical/preclinical contentions and fallacies of evidence. Am. J. Physiol. Endocrinol. Metab..

[104] McElhinny A.S., Kakinuma K., Sorimachi H., Labeit S., Gregorio C.C. (2002). Muscle-specific RING finger-1 interacts with titin to regulate sarcomeric M-line and thick filament structure and may have nuclear functions via its interaction with glucocorticoid modulatory element binding protein-1. J. Cell Biol..

[105] Chen S.N., Czernuszewicz G., Tan Y., Lombardi R., Jin J., Willerson J.T., Marian A.J. (2012). Human molecular genetic and functional studies identify TRIM63, encoding Muscle RING Finger Protein 1, as a novel gene for human hypertrophic cardiomyopathy. Circ. Res..

[106] Cohen S., Brault J.J., Gygi S.P., Glass D.J., Valenzuela D.M., Gartner C., Latres E., Goldberg A.L. (2009). During muscle atrophy, thick, but not thin, filament components are degraded by MuRF1-dependent ubiquitylation. J. Cell Biol..

[107] Arya R., Kedar V., Hwang J.R., McDonough H., Li H.-H., Taylor J., Patterson C. (2004). Muscle ring finger protein-1 inhibits PKC{epsilon} activation and prevents cardiomyocyte hypertrophy. J. Cell Biol..

[108] Savarese M., Jonson P.H., Huovinen S., Paulin L., Auvinen P., Udd B., Hackman P. (2018). The complexity of titin splicing pattern in human adult skeletal muscles. Skelet. Muscle.

[109] Lange S., Xiang F., Yakovenko A., Vihola A., Hackman P., Rostkova E., Kristensen J., Brandmeier B., Franzen G., Hedberg B. (2005). The kinase domain of titin controls muscle gene expression and protein turnover. Science.

[110] Olivé M., Abdul-Hussein S., Oldfors A., González-Costello J., van der Ven P.F.M., Fürst D.O., González L., Moreno D., Torrejón-Escribano B., Alió J. (2015). New cardiac and skeletal protein aggregate myopathy associated with combined MuRF1 and MuRF3 mutations. Hum. Mol. Genet..

[111] Hwee D.T., Baehr L.M., Philp A., Baar K., Bodine S.C. (2014). Maintenance of muscle mass and load-induced growth in Muscle RING Finger 1 null mice with age. Aging Cell.

[112] Labeit S., Kohl C.H., Witt C.C., Labeit D., Jung J., Granzier H. (2010). Modulation of muscle atrophy, fatigue and MLC phosphorylation by MuRF1 as indicated by hindlimb suspension studies on MuRF1-KO mice. J. Biomed. Biotechnol..

[113] Sartori R., Schirwis E., Blaauw B., Bortolanza S., Zhao J., Enzo E., Stantzou A., Mouisel E., Toniolo L., Ferry A. (2013). BMP signaling controls muscle mass. Nat. Genet..

[114] Paul P.K., Bhatnagar S., Mishra V., Srivastava S., Darnay B.G., Choi Y., Kumar A. (2012). The E3 ubiquitin ligase TRAF6 intercedes in starvation-induced skeletal muscle atrophy through multiple mechanisms. Mol. Cell. Biol..

[115] Nagpal P., Plant P.J., Correa J., Bain A., Takeda M., Kawabe H., Rotin D., Bain J.R., Batt J.A.E. (2012). The ubiquitin ligase Nedd4-1 participates in denervation-induced skeletal muscle atrophy in mice. PLoS ONE.

[116] An C.-I., Ganio E., Hagiwara N. (2013). Trip12, a HECT domain E3 ubiquitin ligase, targets Sox6 for proteasomal degradation and affects fiber type-specific gene expression in muscle cells. Skelet. Muscle.

[117] Clarke B.A., Drujan D., Willis M.S., Murphy L.O., Corpina R.A., Burova E., Rakhilin S.V., Stitt T.N., Patterson C., Latres E. (2007). The E3 Ligase MuRF1 Degrades Myosin Heavy Chain Protein in Dexamethasone-Treated Skeletal Muscle. Cell Metab..

[118] Baehr L.M., Furlow J.D., Bodine S.C. (2011). Muscle sparing in muscle RING finger 1 null mice: Response to synthetic glucocorticoids. J. Physiol..

[119] Shoji S., Pennington R.J. (1977). The effect of cortisone on protein breakdown and synthesis in rat skeletal muscle. Mol. Cell. Endocrinol..

[120] Maejima Y., Usui S., Zhai P., Takamura M., Kaneko S., Zablocki D., Yokota M., Isobe M., Sadoshima J. (2014). Muscle-specific RING finger 1 negatively regulates pathological cardiac hypertrophy through downregulation of calcineurin A. Circ. Heart Fail..

[121] Willis M.S., Ike C., Li L., Wang D.-Z., Glass D.J., Patterson C. (2007). Muscle ring finger 1, but not muscle ring finger 2, regulates cardiac hypertrophy in vivo. Circ. Res..

[122] Willis M.S., Schisler J.C., Li L., Rodríguez J.E., Hilliard E.G., Charles P.C., Patterson C. (2009). Cardiac muscle ring finger-1 increases susceptibility to heart failure in vivo. Circ. Res..

[123] Willis M.S., Parry T.L., Brown D.I., Mota R.I., Huang W., Beak J.Y., Sola M., Zhou C., Hicks S.T., Caughey M.C. (2019). Doxorubicin Exposure Causes Subacute Cardiac Atrophy Dependent on the Striated Muscle-Specific Ubiquitin Ligase MuRF1. Circ. Heart Fail..

[124] Verdecchia P., Angeli F., Borgioni C., Gattobigio R., de Simone G., Devereux R.B., Porcellati C. (2003). Changes in cardiovascular risk by reduction of left ventricular mass in hypertension: A meta-analysis. Am. J. Hypertens..

[125] Conraads V.M., Vrints C.J., Rodrigus I.E., Hoymans V.Y., van Craenenbroeck E.M., Bosmans J., Claeys M.J., van Herck P., Linke A., Schuler G. (2010). Depressed expression of MuRF1 and MAFbx in areas remote of recent myocardial infarction: A mechanism contributing to myocardial remodeling?. Basic Res. Cardiol..

[126] Jokela M., Baumann P., Huovinen S., Penttilä S., Udd B. (2019). Homozygous Nonsense Mutation p.Q274X in TRIM63 (MuRF1) in a Patient with Mild Skeletal Myopathy and Cardiac Hypertrophy. J. Neuromuscul. Dis..

[127] Hirner S., Krohne C., Schuster A., Hoffmann S., Witt S., Erber R., Sticht C., Gasch A., Labeit S., Labeit D. (2008). MuRF1-dependent regulation of systemic carbohydrate metabolism as revealed from transgenic mouse studies. J. Mol. Biol..

[128] Wallimann T., Tokarska-Schlattner M., Schlattner U. (2011). The creatine kinase system and pleiotropic effects of creatine. Amino Acids.

[129] Fielitz J., Kim M.-S., Shelton J.M., Latif S., Spencer J.A., Glass D.J., Richardson J.A., Bassel-Duby R., Olson E.N. (2007). Myosin accumulation and striated muscle myopathy result from the loss of muscle RING finger 1 and 3. J. Clin. Investig..

[130] Eble D.M., Spragia M.L., Ferguson A.G., Samarel A.M. (1999). Sarcomeric myosin heavy chain is degraded by the proteasome. Cell Tissue Res..

[131] Ikemoto M., Nikawa T., Takeda S., Watanabe C., Kitano T., Baldwin K.M., Izumi R., Nonaka I., Towatari T., Teshima S. (2001). Space shuttle flight (STS-90) enhances degradation of rat myosin heavy chain in association with activation of ubiquitin-proteasome pathway. FASEB J..

[132] Mearini G., Gedicke C., Schlossarek S., Witt C.C., Krämer E., Cao P., Gomes M.D., Lecker S.H., Labeit S., Willis M.S. (2010). Atrogin-1 and MuRF1 regulate cardiac MyBP-C levels via different mechanisms. Cardiovasc. Res..

[133] Haus J.M., Carrithers J.A., Carroll C.C., Tesch P.A., Trappe T.A. (2007). Contractile and connective tissue protein content of human skeletal muscle: Effects of 35 and 90 days of simulated microgravity and exercise countermeasures, American Journal of Physiology-Regulatory. Integr. Comp. Physiol..

[134] Borina E., Pellegrino M.A., D’Antona G., Bottinelli R. (2010). Myosin and actin content of human skeletal muscle fibers following 35 days bed rest. Scand. J. Med. Sci. Sports.

[135] Cosper P.F., Leinwand L.A. (2012). Myosin heavy chain is not selectively decreased in murine cancer cachexia. Int. J. Cancer.

[136] Polge C., Heng A.-E., Jarzaguet M., Ventadour S., Claustre A., Combaret L., Bechet D., Matondo M., Uttenweiler-Joseph S., Monsarrat B. (2011). Muscle actin is polyubiquitinylated in vitro and in vivo and targeted for breakdown by the E3 ligase MuRF1. FASEB J..

[137] Ventadour S., Jarzaguet M., Wing S.S., Chambon C., Combaret L., Béchet D., Attaix D., Taillandier D. (2007). A new method of purification of proteasome substrates reveals polyubiquitination of 20 S proteasome subunits. J. Biol. Chem..

[138] Polge C., Cabantous S., Deval C., Claustre A., Hauvette A., Bouchenot C., Aniort J., Béchet D., Combaret L., Attaix D. (2018). A muscle-specific MuRF1-E2 network requires stabilization of MuRF1-E2 complexes by telethonin, a newly identified substrate. J. Cachexia Sarcopenia Muscle.

[139] Vainzof M., Moreira E.S., Suzuki O.T., Faulkner G., Valle G., Beggs A.H., Carpen O., Ribeiro A.F., Zanoteli E., Gurgel-Gianneti J. (2002). Telethonin protein expression in neuromuscular disorders. Biochim. Biophys. Acta Mol. Basis Dis..

[140] Bos J.M., Poley R.N., Ny M., Tester D.J., Xu X., Vatta M., Towbin J.A., Gersh B.J., Ommen S.R., Ackerman M.J. (2006). Genotype—phenotype relationships involving hypertrophic cardiomyopathy-associated mutations in titin, muscle LIM protein, and telethonin. Mol. Genet. Metab..

[141] De la Hoz C.P.de., Hernández-Laín A., Olivé M., Fernández-Marmiesse A., Domínguez-González C. (2016). Novel mutation in TCAP manifesting with asymmetric calves and early-onset joint retractions. Neuromuscul. Disord..

[142] Knöll R., Linke W.A., Zou P., Miocic S., Kostin S., Buyandelger B., Ku C.-H., Neef S., Bug M., Schäfer K. (2011). Telethonin deficiency is associated with maladaptation to biomechanical stress in the mammalian heart. Circ. Res..

[143] Knöll R., Hoshijima M., Hoffman H.M., Person V., Lorenzen-Schmidt I., Bang M.-L., Hayashi T., Shiga N., Yasukawa H., Schaper W. (2002). The Cardiac Mechanical Stretch Sensor Machinery Involves a Z Disc Complex that Is Defective in a Subset of Human Dilated Cardiomyopathy. Cell.

[144] Nicholas G., Thomas M., Langley B., Somers W., Patel K., Kemp C.F., Sharma M., Kambadur R. (2002). Titin-cap associates with, and regulates secretion of, Myostatin. J. Cell. Physiol..

[145] Kojic S., Medeot E., Guccione E., Krmac H., Zara I., Martinelli V., Valle G., Faulkner G. (2004). The Ankrd2 protein, a link between the sarcomere and the nucleus in skeletal muscle. J. Mol. Biol..

[146] Frey N., Olson E.N. (2002). Calsarcin-3, a novel skeletal muscle-specific member of the calsarcin family, interacts with multiple Z-disc proteins. J. Biol. Chem..

[147] Heng A.E., Ventadour S., Jarzaguet M., Pouch-Pélissier M.N., Guezennec C.Y., Bigard X., Attaix D., Taillandier D. (2008). Coordinate expression of the 19S regulatory complex and evidence for ubiquitin-dependent telethonin degradation in the unloaded soleus muscle. Int. J. Biochem. Cell Biol..

[148] Wallimann T., Schlösser T., Eppenberger H.M. (1984). Function of M-line-bound creatine kinase as intramyofibrillar ATP regenerator at the receiving end of the phosphorylcreatine shuttle in muscle. J. Biol. Chem..

[149] Ventura-Clapier R., Veksler V., Hoerter J.A. (1994). Myofibrillar creatine kinase and cardiac contraction. Mol. Cell. Biochem..

[150] Huttlin E.L., Jedrychowski M.P., Elias J.E., Goswami T., Rad R., Beausoleil S.A., Villén J., Haas W., Sowa M.E., Gygi S.P. (2010). A Tissue-Specific Atlas of Mouse Protein Phosphorylation and Expression. Cell.

[151] Lin G., Liu Y., MacLeod K.M. (2009). Regulation of muscle creatine kinase by phosphorylation in normal and diabetic hearts. Cell. Mol. Life Sci..

[152] Dieni C.A., Storey K.B. (2009). Creatine kinase regulation by reversible phosphorylation in frog muscle. Comp. Biochem. Physiol. B Biochem. Mol. Biol..

[153] Zhao T.-J., Yan Y.-B., Liu Y., Zhou H.-M. (2007). The generation of the oxidized form of creatine kinase is a negative regulation on muscle creatine kinase. J. Biol. Chem..

[154] Nakagawa T., Tsuruma K., Uehara T., Nomura Y. (2008). GMEB1, a novel endogenous caspase inhibitor, prevents hypoxia- and oxidative stress-induced neuronal apoptosis. Neurosci. Lett..

[155] Wanders R.J.A., Duran M., Loupatty F.J. (2012). Enzymology of the branched-chain amino acid oxidation disorders: The valine pathway. J. Inherit. Metab. Dis..

[156] Oka T., Dai Y.-S., Molkentin J.D. (2005). Regulation of calcineurin through transcriptional induction of the calcineurin a beta promoter in vitro and in vivo. Mol. Cell. Biol..

[157] Rothermel B.A., McKinsey T.A., Vega R.B., Nicol R.L., Mammen P., Yang J., Antos C.L., Shelton J.M., Bassel-Duby R., Olson E.N. (2001). Myocyte-enriched calcineurin-interacting protein, MCIP1, inhibits cardiac hypertrophy in vivo. Proc. Natl. Acad. Sci. USA.

[158] Heineke J., Auger-Messier M., Correll R.N., Xu J., Benard M.J., Yuan W., Drexler H., Parise L.V., Molkentin J.D. (2010). CIB1 is a regulator of pathological cardiac hypertrophy. Nat. Med..

[159] Heineke J., Molkentin J.D. (2006). Regulation of cardiac hypertrophy by intracellular signalling pathways. Nat. Rev. Mol. Cell Biol..

[160] Molkentin J.D. (2004). Calcineurin–NFAT signaling regulates the cardiac hypertrophic response in coordination with the MAPKs. Cardiovasc. Res..

[161] Ferrandi C., Ballerio R., Gaillard P., Giachetti C., Carboni S., Vitte P.-A., Gotteland J.-P., Cirillo R. (2004). Inhibition of c-Jun N-terminal kinase decreases cardiomyocyte apoptosis and infarct size after myocardial ischemia and reperfusion in anaesthetized rats. Br. J. Pharmacol..

[162] Milano G., Morel S., Bonny C., Samaja M., von Segesser L.K., Nicod P., Vassalli G. (2007). A peptide inhibitor of c-Jun NH2-terminal kinase reduces myocardial ischemia-reperfusion injury and infarct size in vivo. Am. J. Physiol. Heart Circ. Physiol..

[163] Maillet M., van Berlo J.H., Molkentin J.D. (2013). Molecular basis of physiological heart growth: Fundamental concepts and new players. Nat. Rev. Mol. Cell Biol..

[164] Wadosky K.M., Rodríguez J.E., Hite R.L., Min J., Walton B.L., Willis M.S. (2014). Muscle RING finger-1 attenuates IGF-I-dependent cardiomyocyte hypertrophy by inhibiting JNK signaling. Am. J. Physiol. Endocrinol. Metab..

[165] Zhao M., Chow A., Powers J., Fajardo G., Bernstein D. (2004). Microarray analysis of gene expression after transverse aortic constriction in mice. Physiol. Genom..

[166] Rudolf R., Bogomolovas J., Strack S., Choi K.-R., Khan M.M., Wagner A., Brohm K., Hanashima A., Gasch A., Labeit D. (2013). Regulation of nicotinic acetylcholine receptor turnover by MuRF1 connects muscle activity to endo/lysosomal and atrophy pathways. Age.

[167] Kjaerulff O., Brodin L., Jung A. (2011). The structure and function of endophilin proteins. Cell Biochem. Biophys..

[168] Li J., Barylko B., Eichorst J.P., Mueller J.D., Albanesi J.P., Chen Y. (2016). Association of Endophilin B1 with Cytoplasmic Vesicles. Biophys. J..

[169] Takahashi Y., Coppola D., Matsushita N., Cualing H.D., Sun M., Sato Y., Liang C., Jung J.U., Cheng J.Q., Mulé J.J. (2007). Bif-1 interacts with Beclin 1 through UVRAG and regulates autophagy and tumorigenesis. Nat. Cell Biol..

[170] Khan M.M., Strack S., Wild F., Hanashima A., Gasch A., Brohm K., Reischl M., Carnio S., Labeit D., Sandri M. (2014). Role of autophagy, SQSTM1, SH3GLB1, and TRIM63 in the turnover of nicotinic acetylcholine receptors. Autophagy.

[171] Li G., Ji T., Chen J., Fu Y., Hou L., Feng Y., Zhang T., Song T., Zhao J., Endo Y. (2017). CRL4DCAF8 Ubiquitin Ligase Targets Histone H3K79 and Promotes H3K9 Methylation in the Liver. Cell Rep..

[172] Nowak M., Suenkel B., Porras P., Migotti R., Schmidt F., Kny M., Zhu X., Wanker E.E., Dittmar G., Fielitz J. (2019). DCAF8, a novel MuRF1 interaction partner, promotes muscle atrophy. J. Cell Sci..

[173] Pozzi A., Ibanez M.R., Gatica A.E., Yang S., Wei S., Mei S., Falck J.R., Capdevila J.H. (2007). Peroxisomal proliferator-activated receptor-alpha-dependent inhibition of endothelial cell proliferation and tumorigenesis. J. Biol. Chem..

[174] Rodríguez J.E., Liao J.-Y., He J., Schisler J.C., Newgard C.B., Drujan D., Glass D.J., Frederick C.B., Yoder B.C., Lalush D.S. (2015). The ubiquitin ligase MuRF1 regulates PPARα activity in the heart by enhancing nuclear export via monoubiquitination. Mol. Cell. Endocrinol..

[175] Duff D.A. (2017). Long, Roles for RACK1 in cancer cell migration and invasion. Cell. Signal.

[176] Mochly-Rosen D., Wu G., Hahn H., Osinska H., Liron T., Lorenz J.N., Yatani A., Robbins J., Dorn G.W. (2000). Cardiotrophic effects of protein kinase C epsilon: Analysis by in vivo modulation of PKCepsilon translocation. Circ. Res..

[177] Mochly-Rosen D. (1995). Localization of protein kinases by anchoring proteins: A theme in signal transduction. Science.

[178] Gregorio C.C., Granzier H., Sorimachi H., Labeit S. (1999). Muscle assembly: A titanic achievement?. Curr. Opin. Cell Biol..

[179] Machado C., Andrew D.J. (2000). D-Titin: A giant protein with dual roles in chromosomes and muscles. J. Cell Biol..

[180] Obermann W.M., Gautel M., Weber K., Fürst D.O. (1997). Molecular structure of the sarcomeric M band: Mapping of titin and myosin binding domains in myomesin and the identification of a potential regulatory phosphorylation site in myomesin. EMBO J..

[181] Higashikuse Y., Mittal N., Arimura T., Yoon S.H., Oda M., Enomoto H., Kaneda R., Hattori F., Suzuki T., Kawakami A. (2019). Perturbation of the titin/MURF1 signaling complex is associated with hypertrophic cardiomyopathy in a fish model and in human patients. Dis. Models Mech..

[182] Brückner A., Polge C., Lentze N., Auerbach D., Schlattner U. (2009). Yeast Two-Hybrid, a Powerful Tool for Systems Biology. Int. J. Mol. Sci..

[183] Stewart M.D., Ritterhoff T., Klevit R.E., Brzovic P.S. (2016). E2 enzymes: More than just middle men. Cell Res..

[184] Cécile P., Julien A., Andrea A., Agnès C., Cécile C.-G., Clara T., Christiane D., Lydie C., Daniel B., Marco S. (2018). UBE2E1 Is Preferentially Expressed in the Cytoplasm of Slow-Twitch Fibers and Protects Skeletal Muscles from Exacerbated Atrophy upon Dexamethasone Treatment. Cells.

[185] Marblestone J.G., Butt S., McKelvey D.M., Sterner D.E., Mattern M.R., Nicholson B., Eddins M.J. (2013). Comprehensive ubiquitin E2 profiling of ten ubiquitin E3 ligases. Cell Biochem. Biophys..

[186] Van Wijk S.J.L., de Vries S.J., Kemmeren P., Huang A., Boelens R., Bonvin A.M.J.J., Timmers H.T.M. (2009). A comprehensive framework of E2-RING E3 interactions of the human ubiquitin-proteasome system. Mol. Syst. Biol..

[187] Markson G., Kiel C., Hyde R., Brown S., Charalabous P., Bremm A., Semple J., Woodsmith J., Duley S., Salehi-Ashtiani K. (2009). Analysis of the human E2 ubiquitin conjugating enzyme protein interaction network. Genome Res..

[188] Napolitano L.M., Jaffray E.G., Hay R.T., Meroni G. (2011). Functional interactions between ubiquitin E2 enzymes and TRIM proteins. Biochem. J..

[189] Banerjee R., He J., Spaniel C., Quintana M.T., Wang Z., Bain J., Newgard C.B., Muehlbauer M.J., Willis M.S. (2015). Non-targeted metabolomics analysis of cardiac Muscle Ring Finger-1 (MuRF1), MuRF2, and MuRF3 in vivo reveals novel and redundant metabolic changes. Metabolomics.

[190] Attaix D., Ventadour S., Codran A., Béchet D., Taillandier D., Combaret L. (2005). The ubiquitin-proteasome system and skeletal muscle wasting. Essays Biochem..

[191] Jagoe R.T., Goldberg A.L. (2001). What do we really know about the ubiquitin-proteasome pathway in muscle atrophy?. Curr. Opin. Clin. Nutr. Metab. Care.

[192] Eddins M.J., Marblestone J.G., Kumar K.G.S., Leach C.A., Sterner D.E., Mattern M.R., Nicholson B. (2011). Targeting the ubiquitin E3 ligase MuRF1 to inhibit muscle atrophy. Cell Biochem. Biophys..

[193] Adams V., Bowen T.S., Werner S., Barthel P., Amberger C., Konzer A., Graumann J., Sehr P., Lewis J., Provaznik J. (2019). Small-molecule-mediated chemical knock-down of MuRF1/MuRF2 and attenuation of diaphragm dysfunction in chronic heart failure. J. Cachexia Sarcopenia Muscle.

